# From Semantic Modeling to Precision Radiotherapy: An AI Framework Linking Radiobiology, Oncology, and Public Health Integration

**DOI:** 10.3390/biomedicines13122862

**Published:** 2025-11-24

**Authors:** Fernando Gomes de Souza Jr., José Maria Aliaga Jr., Paulo C. Duarte Jr., Shirley Crispilho, Carolina Delfino, Daniele Brandão, Fernando Zamprogno e Silva

**Affiliations:** 1 Instituto de Macromoléculas Professora Eloisa Mano, Universidade Federal do Rio de Janeiro (UFRJ), Centro de Tecnologia, Cidade Universitária, Rio de Janeiro 21941-598, Brazil; 2 Programa de Engenharia da Nanotecnologia—COPPE, Universidade Federal do Rio de Janeiro (UFRJ), Centro de Tecnologia, Cidade Universitária, Rio de Janeiro 21941-972, Brazil; carolina.c.delfino@gmail.com (C.D.); danielesb29@gmail.com (D.B.); 3 Department of Electrical and Computer Engineering, Florida International University (FIU), 10555 West Flagler Street, EC3900, Miami, FL 33174, USA; 4 Departamento de Oncologia, Hospital Paulistano, São Paulo 01321-001, Brazil; 5 Departamento de Oncologia, Prevent Senior, São Paulo 01401-001, Brazil; 6 Bioengineering Department, Institute Dante Pazzanese of Cardiology, São Paulo 04012-909, Brazil; paulo.duarte@dantepazzanese.org.br (P.C.D.J.); crispilhoshirleyferraz@gmail.com (S.C.); 7 Department of Civil Construction, State University of Londrina, Londrina 86057-970, Parana, Brazil; 8 Department of Oncology, Hospital Meridional S.A., Vila Velha 29101-295, Espírito Santo, Brazil

**Keywords:** AI-based semantic and temporal analysis, co-occurrence supergraph, clinical-anatomical axis, mechanistic-molecular axis, DNA repair and molecular response, advanced radiotherapy technologies (FLASH-RT, hadron therapy, voxel-level analytics)

## Abstract

**Background/Objectives:** Radiotherapy, radiobiology, and oncology have evolved rapidly over the past six decades. This progress has generated vast but fragmented bodies of scientific evidence. The present study aimed to systematically map and interpret their conceptual and temporal development using artificial intelligence (AI)-based methods. It highlights the integration between molecular mechanisms, clinical applications, and technological innovation within a precision radiotherapy framework. **Methods:** A corpus of 3343 unique articles (1964–2025) was retrieved from Scopus, PubMed, and Web of Science. Records were harmonized through deduplication, lemmatization, and metadata normalization. Topic modeling using Latent Dirichlet Allocation (LDA) and co-occurrence network analysis were applied to identify dominant research axes. Semantic and temporal analyses were conducted to reveal patterns, emerging trends, and translational connections across decades. **Results:** Three historical phases were identified. The first was a period of limited production (1964–1990). The second showed moderate growth (1991–2010). The third, from 2011 to 2024, represented exponential expansion, with publication peaks in 2020 and 2023. LDA revealed two principal axes. The first, a clinical–anatomical axis, focused on cancer sites, treatment modalities, and prognosis. The second, a mechanistic–molecular axis, centered on DNA repair, radiosensitivity, and biomarkers. Case synthesis from 2014–2025 defined five operational classes: DNA repair and molecular response; precision oncology and genomic modeling; individual radiosensitivity; mechanisms of radioresistance; and advanced technologies such as FLASH radiotherapy and optimized brachytherapy. **Conclusions:** AI-driven semantic and temporal analyses showed that radiotherapy has matured into an interconnected and interdisciplinary domain. The derived Precision Radiotherapy Implementation Plan translates molecular and computational insights into clinically actionable strategies. These approaches can enhance survival, reduce toxicity, and inform equitable health policies for advanced cancer care.

## 1. Introduction

Over the past decades, scientific research has grown exponentially, generating vast reservoirs of knowledge across numerous fields [1,2,3,4]. Yet, a large fraction of that knowledge remains underutilized. Much of it is scattered across databases and difficult to access or interpret, especially in highly specialized domains [5,6,7,8,9].

This study is based on a central premise: the combination of literature mining and artificial intelligence (AI) can transform how we map the thematic evolution and conceptual foundations of radiotherapy [10,11,12,13,14,15,16,17,18,19,20,21,22,23,24,25,26,27,28], radiobiology [29,30,31,32,33,34,35,36,37,38,39,40,41,42,43,44,45,46,47,48,49,50,51,52], and oncology [53,54,55,56,57,58,59,60,61,62,63,64,65,66,67,68,69,70,71,72]. Automated, scalable, and interpretable analyses can overcome the limitations of traditional searches and manual curation. As a result, this approach provides a broader, more current, and interdisciplinary understanding of the state of the art [7,73,74,75].

Traditional bibliometric tools often fail to capture subtle but significant patterns in specialized areas. This limitation underscores the value of AI-based methods that exploit metadata, semantic models, and co-occurrence networks. In previous work, our use of unsupervised machine learning and text mining revealed emerging trends and knowledge gaps in fields such as nanocomposites, controlled drug delivery, photovoltaics, catalysis, biosynthesis, and spectroscopy. These results demonstrated that computational approaches can accelerate the synthesis and application of scientific knowledge [76,77,78,79,80,81,82,83,84,85,86,87,88,89,90,91,92,93].

In the present work, we apply this strategy to an interdisciplinary corpus of thousands of articles indexed in Scopus, PubMed, and Web of Science. Our goal is to rigorously structure, validate, and interpret how radiotherapy, radiobiology, and oncology have evolved as an interconnected domain from 1964 to 2025. To our knowledge, this represents the first comprehensive effort to apply AI-based semantic and temporal analyses to these disciplines at such scale and depth.

None of the existing studies have jointly examined the semantic and temporal dimensions of the literature through a unified analysis pipeline. A clear methodological gap persists between bibliometric or quantitative approaches and clinically oriented predictive models. Current research can be grouped into four main strands, each addressing specific aspects of the problem. Bibliometric and visualization studies, for example, often rely on tools such as VOSviewer (version 1.6.20) and CiteSpace (version 6.4.R2) to explore keyword evolution and co-authorship networks. Wang et al. (2025) [94] reported a keyword co-occurrence network that illustrates major field trends between 2014 and 2025. However, such studies remain mostly descriptive and rarely include topic modeling, deep semantic analysis, or time-series methods.

Methodological proposals like that of Andrei and Arandjelovic (2016) [95] employ hierarchical Dirichlet processes and temporal similarity graphs to model topic evolution. Yet, these frameworks have not been applied in biomedical contexts or in radiotherapy. Meanwhile, studies such as those by Tabibi et al. (2025) [96] and Lastrucci et al. (2024) [97] use deep learning to predict clinical outcomes and refine radiotherapy strategies. This has improved treatment precision and efficacy. However, these works focus on clinical endpoints rather than on mining or semantically representing the scientific literature itself.

Latent Dirichlet Allocation (LDA) remains a widely adopted method for uncovering hidden topics within large text corpora. In LDA, each document is represented as a mixture of topics, and each word is associated with one topic with a certain probability [98,99]. This enables the automatic discovery of meaningful patterns in large datasets. Because of its scalability and interpretability, LDA is extensively used in text mining, information retrieval, and bibliometrics.

The performance of LDA depends strongly on the number of topics and metrics such as coherence and perplexity. To improve its performance, several hybrid and dynamic variants have been developed. Currently, Gibbs sampling is the most common inference method. More recent extensions—including semantic similarity, contextual embeddings such as BERT [100], and variational Bayesian inference for large datasets—have enhanced the interpretability and robustness of LDA. Nonetheless, the method has limitations, such as sensitivity to initialization and difficulty in modeling complex linguistic relationships. Despite these issues, LDA remains the cornerstone of semantic modeling. It enables AI-based frameworks like the one used here to transform fragmented scientific evidence into structured, actionable knowledge [101,102,103].

Several reviews, including those by Momin et al. (2021) [104] and Trifiletti and Showalter (2015) [105], discuss the integration of clinical and genomic data in radiotherapy. However, they do not include semantic or temporal analyses of the scientific output. Taken together, these strands of literature highlight a methodological gap between purely bibliometric studies and clinically oriented predictive models. To our knowledge, no published work has combined semantic modeling (e.g., topic modeling and co-occurrence networks) with structured temporal analysis (e.g., time-series and change-point detection) in a unified framework for radiotherapy, radiobiology, and oncology. This absence justifies the novelty of the present study, which aims to integrate bibliometric indicators, semantic modeling, and temporal dynamics into a single framework that offers both analytical insight and predictive perspective on the field’s evolution.

By integrating major databases and processing over 3000 unique peer-reviewed articles from Scopus, PubMed, and Web of Science—excluding editorials, commentaries, and non-scientific records—this study employs advanced computational modeling to move beyond traditional bibliometrics. The result is a dynamic and interpretable view of how these fields have evolved over six decades, linking molecular mechanisms, clinical practice, and technological development within a unified analytical framework.

In essence, this paper introduces a new approach to accelerate scientific progress. It uses AI to connect and integrate dispersed research into a coherent body of knowledge. Instead of allowing discoveries to remain fragmented, AI transforms them into unified and actionable insights. This accelerates translational research and promotes more efficient, equitable, and effective healthcare.

## 2. Methods

The search strategies applied to Scopus, PubMed, and Web of Science were carefully harmonized to identify publications addressing the intersection of radiotherapy, radiobiology, and oncology. The guiding question was: “What are the key connections and advances at the interface of radiotherapy, radiobiology, and oncology as reflected in the recent scientific literature?”

The query used in Scopus was TITLE-ABS-KEY ((radiotherapy) AND (radiobiology OR “radiation biology”) AND (oncology)).

In PubMed, a combination of MeSH terms and free-text fields was applied—(“Radiotherapy”[MeSH Terms] OR “Radiotherapy”[All Fields]) AND (“Radiobiology”[All Fields] OR “Radiation Biology”[All Fields]) AND (“Oncology”[MeSH Terms] OR “Oncology”[All Fields]).

In Web of Science, the direct key (Radiotherapy AND Radiobiology AND Oncology) was used.

These strategies initially retrieved 2507 records from PubMed, 741 from Scopus, and 714 from Web of Science. The total combined corpus contained 3962 articles.

All analyses were performed in Python 3.10 (Python Software Foundation, Beaverton, OR, USA) on Ubuntu 22.04 LTS, using a workstation with 64 GB RAM and an NVIDIA RTX A5000 GPU. The computational pipeline consisted entirely of open-source libraries and custom research software.

We used pandas (2.2.2), numpy (1.26.4), and matplotlib (3.8.4) for data handling, statistics, and visualization. For semantic modeling, we employed gensim (4.3.1) to train Latent Dirichlet Allocation (LDA) models.

Text preprocessing was conducted with NLTK (3.9.1) for tokenization, lemmatization, and stopword filtering.

Lexical salience visualization was performed using WordCloud (1.9.3), and Gephi (0.10.1; University of Paris, France) was used for the calculation of network metrics.

We augmented this stack with an integrated suite of research tools developed by Fernando Gomes (UFRJ, 2025 [106,107,108,109,110]). These included WordSpectrum (longitudinal textual analysis and visualization of term trajectories), SLAT (bibliometric profiling and trend mapping via LDA topic modeling and sentiment analysis), VOSDataAnalyzer (quantification of co-occurrence structures and generation of VOSviewer-compatible outputs, including Total Link Strength and Euclidean distance metrics), BiDAVis (keyword clustering and network structure visualization using LSBI computations), and PaperProcessor (automated PDF ingestion, OCR [PyMuPDF, Tesseract], NLP-based summarization, and topic modeling).

The PaperProcessor module integrated large language models (LLaMA 3 [8B], GPT-4o) with cosine-similarity checks to validate AI-generated summaries. The computational workflow automatically retrieved, deduplicated, and normalized publication metadata from Scopus, PubMed, and Web of Science.

After harmonizing the major bibliographic fields (Title, Abstract, DOI, Year, and Source), we performed semantic preprocessing, statistical normalization, and topic modeling. Independent LDA models were trained for titles and abstracts to delimit thematic clusters and extract representative terms. From these, we conducted analyses of temporal dynamics, word frequency distributions, and network co-occurrence patterns.

Keyword-based graphs and integrated supergraphs were constructed to capture the global connectivity between research fields. Internal validation of AI-derived clusters was performed through cosine similarity to evaluate semantic coherence. This ensured transparency, reproducibility, and extensibility for future meta-analyses and external cross-validation.

When divergences occurred between title-based and abstract-based models, concordance was achieved by cross-checking the top 20 keywords from each model. Only terms that appeared in both or presented consistent thematic alignment (cosine similarity > 0.5) were retained. This procedure ensured semantic robustness and reproducibility while minimizing corpus-level variance between document sections.

The current study used only publicly available bibliographic and metadata records from Scopus, PubMed, and Web of Science. It did not use human subjects, patient data, or identifiable personal information. Thus, institutional review board (IRB) approval was not required.

The temporal coverage of the corpus spans six decades, from January 1964 to March 2025, encompassing the complete historical trajectory of research in radiotherapy, radiobiology, and oncology indexed in the three databases. Only peer-reviewed journal articles written in English were included. Non-research items such as editorials, letters, conference abstracts, and commentaries were excluded.

**Figure 1:**
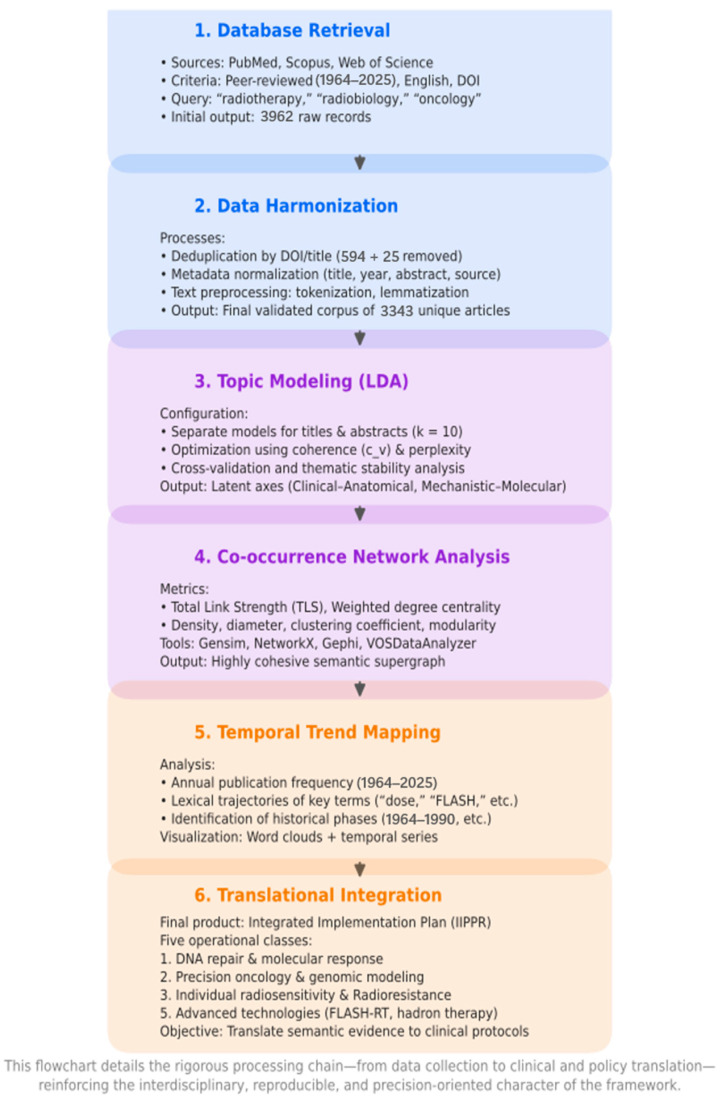
**AI-Driven Methodological Pipeline for Semantic Analysis in Radiotherapy.** This flowchart details the rigorous processing chain—from data collection to clinical and policy translation—reinforcing the interdisciplinary, reproducible, and precision-oriented character of the framework.

Records without DOIs or with incomplete bibliographic metadata were removed during the deduplication process. The final harmonized corpus therefore represents a validated and language-standardized dataset suitable for reproducible, AI-based bibliometric analysis. A summary of the workflow is presented in Figure 1.

## 3. Results

### 3.1. Corpus Construction and Harmonization

The structured computational pipeline began with the automated ingestion of files from Scopus, PubMed, and Web of Science. We standardized and normalized the core data fields. Additional metadata for each source was retained.

The data from all sources were merged. When the same publication appeared more than once, we selected the record containing the most complete information. A total of 594 duplicates were removed based on ID numbers and 25 based on titles. The final dataset contained 3343 unique publications.

Most publications originated from PubMed (66.23%), followed by Scopus (21.96%) and Web of Science (11.82%). The distribution across databases was therefore not uniform.

The five-class framework offers a single paradigm for conceptualizing precision radiotherapy. However, it has not yet been validated using clinical datasets. This study did not aim to model or predict patient-level data. Instead, it focused on organizing and synthesizing evidence at the semantic and structural levels. Validation of this framework against clinical datasets represents a key next step.

**Figure 2:**
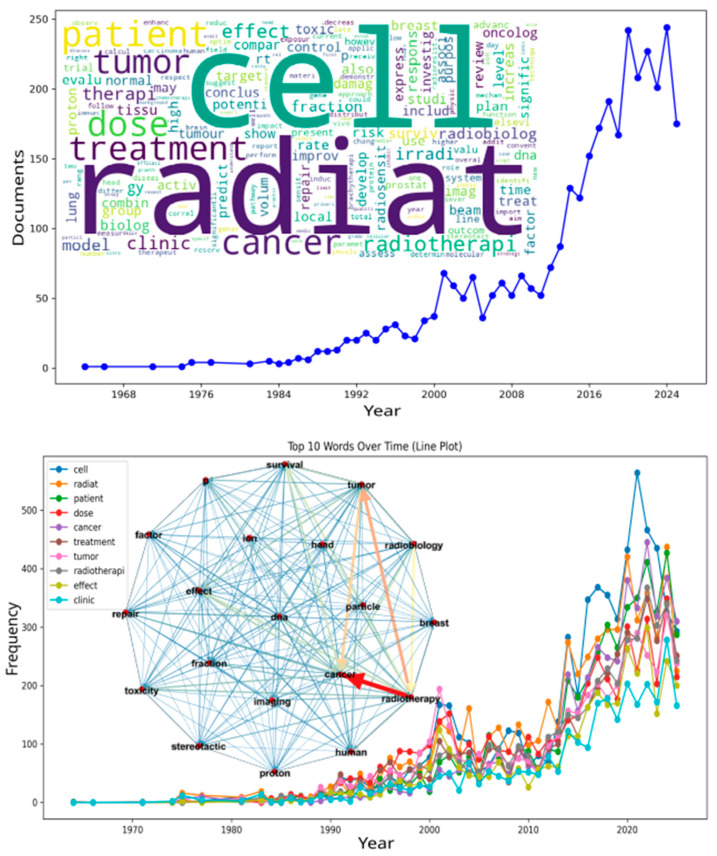
**Word cloud, temporal evolution of publication output, term frequency, and co-occurrence supergraph.** The figure presents multiple layers of lexical evidence from the radiobiology and radiation oncology literature. It provides an overview of how the field has expanded and organized over time. The top panel shows a line plot of the number of publications per year from the 1960s to the present. It reveals a prolonged period of gradual growth followed by a rapid increase during the last decade. The word cloud on the right displays all terms scaled by frequency. The most prominent terms are cell, radiation, patient, dose, treatment, and cancer. These reflect the persistent interplay between mechanistic biology, dosimetry, clinical application, and treatment outcomes. The bottom panel shows the longitudinal trends of the top ten terms. Their steep increase in the 2000s supports the hypothesis of conceptual consolidation in the field. At the center, the co-occurrence supergraph illustrates the relationships among these terms. Each node represents a term, and its size is proportional to its centrality. The edges indicate the relationships between terms. Edge color ranges from blue (weak) to red (strong), and line thickness increases with co-occurrence frequency. Together, these analytical layers depict not only the growth of scientific output but also the semantic structure underlying the evolution of contemporary precision radiotherapy. Cellular mechanisms, clinical endpoints, and technological parameters converge within this framework to drive progress in the discipline.

Figure 2 provides a graphical synthesis of the results. The upper section presents the number of publications per year, showing three historical phases. The first corresponds to a period of low publication rate (1964–1990). The second shows moderate growth (1991–2010). The third period, from 2011 onward, represents rapid growth and marks the beginning of the “Integration Era” (2011–2024).

This recent phase is characterized by paradigm shifts such as radiogenomics-based stratification, adaptive planning, and AI-guided dose optimization. Advances in CT-simulation–guided interventional radiotherapy, dynamic joint predictive modeling, nanotheranostic systems, and oxygen-independent radiodynamic therapy illustrate these transitions [111,112,113,114].

The years 2020 and 2023 recorded the highest publication counts (~240). The apparent decrease in 2025 is likely due to incomplete indexing and may not represent the true publication volume.

The word cloud at the bottom of the figure was generated from titles and abstracts. Common terms such as *radiat*, *cell*, *treatment*, *cancer*, *dose*, *patient*, *tumor*, *radiotherapi*, *fraction*, *therap*, and *radiobiolog* are highlighted.

A co-occurrence supergraph was constructed after topic extraction to measure corpus-level semantic connectivity. Each node represented a keyword. Each edge indicated the co-occurrence frequency of two phrases within a document. Edge thickness and color intensity were proportional to association strength. These values were calculated from normalized co-occurrence counts using the Total Link Strength (TLS) metric with Euclidean distance normalization.

Node size was scaled by weighted degree centrality to represent each term’s connectedness within the network. The Python 3.10 pipeline used open-source modules: NLTK for tokenization, lemmatization, and biomedical stopword filtering; Gensim for topic modeling (LDA); and NetworkX for graph formation and weighting.

The custom research tools VOSDataAnalyzer and BiDAVis were then employed to compute TLS matrices, LSBI-based clustering, and modularity structures compatible with VOSviewer visualization standards. For each LDA keyword, local co-occurrence graphs were generated using NetworkX. These were merged into a composite supergraph via a spring layout (seed = 42, k = 0.3, 200 iterations) and visualized with matplotlib 3.8.4.

All node and edge data were exported as CSV files (*Source*, *Target*, *Weight*; *Id*, *Degree*) for topological analysis in Gephi 0.10.1 (University of Paris, France). High-resolution outputs were automatically generated in PNG and SVG formats.

The resulting supergraph exhibited strong cohesion and low modularity (density = 0.5, diameter ≈ 2, clustering coefficient = 0.5). This indicates a highly connected thematic network for radiation, radiobiology, and cancer. The weighted degree (1962.2) represents the total node strength based on normalized co-occurrence frequencies. The average degree (9.5) reflects the mean number of unweighted connections per node.

This configuration shows that half of all potential ties between terms are present. Any two nodes can be connected in two steps, and half of each node’s neighbors are mutually linked. The high level of interconnection highlights both global integration and local cohesiveness in radiation-related knowledge. It demonstrates the field’s conceptual complexity and depth.

Additional terms such as *proton*, *irradi*, *brachytherapy*, *repair*, *DNA*, *toxicity*, and *response* refer to molecular mechanisms and emerging techniques. In contrast, *surviv*, *risk*, *model*, and *outcome* emphasize prognosis. The terms *clinic*, *trial*, *meta*, and *evalu* indicate the growing prevalence of translational trials.

The lower panel presents the ten most frequent terms across the years. The term *cell* appears most often, with more than 550 occurrences in 2020. This frequency reflects the large volume of cellular and molecular investigations. Terms such as *radiat*, *patient*, *dose*, *cancer*, *treatment*, *tumor*, and *radiotherapi* also increased in parallel between 2015 and 2023. These are closely linked to the central themes of the field.

The terms *effect* and *clinic* rose gradually over time, highlighting an expanding focus on therapeutic effects and clinical practice. Both play an important role in the field’s continued development. After corpus harmonization, topic modeling was applied to uncover the latent thematic structures within the literature.

### 3.2. Topic Modeling and Co-Occurrence Analysis

Collectively, these results indicate limited vocabulary use until the early 1990s. A gradual increase followed, culminating in a sharp expansion after 2010 that correlates with the development and consolidation of the field.

After text preprocessing, Latent Dirichlet Allocation (LDA) was applied to model the latent semantic structure of the corpus. Models were trained using *k* = 10 topics. This value was defined after a preliminary coherence optimization in which candidate models with *k* = 5–15 were compared using the *c_v* coherence score and perplexity minimization to balance interpretability and generalization.

Texts were lemmatized, filtered by biomedical stopwords, and frequency-trimmed (*no_below* = 2–5; *no_above* = 0.5) to ensure vocabulary stability. Each model was trained separately for titles and abstracts using Gensim 4.3.1, with *passes* = 10 and *random_state* = 42. The optimal configuration corresponded to the highest mean coherence (*c_v* = 0.61) and consistent topic reproducibility across ten independent runs.

Validation combined three approaches: (i) coherence and perplexity metrics, (ii) manual inspection of topic–word distributions, and (iii) cross-model stability checks.

The top 20 LDA-derived keywords from each corpus were then used to construct the co-occurrence supergraph, providing a quantitative foundation for thematic mapping. Titles included *head and neck*, *breast*, and *prostate cancer*; *adverse effects*; *image-guided planning*; and *highly conformal treatment*. Abstracts emphasized *DNA damage and repair*; *dose–response modeling*; *clinical trials*; *immunoradiotherapy*; and *normal tissue adverse effects*.

For each topic cluster, word association maps were generated using the 20 most probable terms. These maps were integrated into a comprehensive semantic map that provided a panoramic view of the field.

The main nodes—*radiotherapy*, *tumor*, *DNA repair*, and *adverse effects*—emerged as central structuring elements within the research network. All files (CSV, SVG, PNG) were exported for visualization and topological analysis in Gephi, ensuring transparency, reproducibility, and quantitative interpretability of results.

Yearly word counts and smoothed frequency curves showed sustained growth in key biological and technological concepts. This confirmed the thematic and structural maturation of the radiotherapy–radiobiology–oncology ecosystem.

The results also revealed an increasingly interdisciplinary field linking radiotherapy, radiobiology, and oncology. Reciprocal interactions were observed between technological developments, cellular processes, and clinical practice. The field has therefore become more complex and multidisciplinary.

The approach emphasizes repeatability, transparency, and reproducibility of methods to ensure interpretability and verifiability of findings. This methodological rigor is essential for advancing the field.

In brief, LDA was applied to the titles and abstracts of the 3343 non-duplicate publications. Ten topics were identified for each corpus, reflecting the wide scope of the literature that encompasses radiotherapy, radiobiology, and oncology. Full counts and model parameters are provided in Equations (A1)–(A20) of the Appendix A and Appendix B, which contain a detailed description of the methodology and results.

In the **titles**, topics ranged from clinical and anatomical foci to molecular mechanisms and technical approaches.

- **Topic_0t:** *cancer*, *radiotherapy*, *patient*, *head*, *neck*, *breast*, *prostate*—concentration on specific tumor types treated with radiotherapy, including advanced cases and toxicity.
- **Topic_1t:** *radiobiology*, *clinical*, *oncology*—integration of biological foundations with clinical practice.
- **Topic_2t:** *cancer*, *breast*, *carcinoma*, *prostate*, *esophageal*—comparative studies among tumor types.
- **Topic_3t:** *radiation*, *oncology*, *biology*, *molecular*—mechanisms of radiation action.
- **Topic_4t:** *stereotactic*, *body*, *radiosurgery*, *lung*—SBRT literature in pulmonary neoplasms.
- **Topic_5t:** *radiation*, *beam*, *ion*, *proton*—dose delivery physics and tissue protection.
- **Topic_6t:** *tumor*, *brain*, *model*, *imaging*—modeling and preclinical studies of brain tumors.
- **Topic_7t:** *tumor*, *cell*, *DNA*, *repair*, *pathway*—molecular biology of DNA damage.
- **Topic_8t:** *cell*, *human*, *expression*, *gene*—in vitro experimentation.
- **Topic_9t:** *dose*, *brachytherapy*, *model*, *radiobiological*—dose modeling and brachytherapy.

In the **abstracts**, the thematic structure was more detailed, reflecting greater methodological granularity.

- **Topic_0a:** *model*, *dose*, *imaging*, *flash*—imaging-based modeling and planning for FLASH-RT.
- **Topic_1a:** *cell*, *tumor*, *DNA*, *repair*, *damage*—biological mechanisms of radiation.
- **Topic_2a:** *proton*, *ion*, *RBE*, *particle*—particle therapy literature.
- **Topic_3a:** *patient*, *survival*, *RT*, *surgery*—prognostic clinical studies.
- **Topic_4a:** *toxicity*, *breast*, *risk*, *Gy*, *SBRT*—toxicity in breast cancer treated with precision radiotherapy.
- **Topic_5a:** *trial*, *immunotherapy*, *preclinical*, *targeted*—immunoradiotherapy and combination therapies.
- **Topic_6a:** *expression*, *gene*, *protein*, *blood*—molecular biomarker research.
- **Topic_7a:** *dose*, *Gy*, *plan*, *mouse*—preclinical trials and validation in animal models.
- **Topic_8a:** *clinical*, *therapy*, *oncology*, *development*—institutional or editorial content.
- **Topic_9a:** *dose*, *fraction*, *tissue*, *effect*—dose fractionation studies in normal tissues.

### 3.3. Thematic Axes and Translational Integration

Our analysis yields a two-dimensional thematic space defined by two orthogonal and interrelated axes: a clinical–anatomical axis and a mechanistic–molecular axis. The clinical–anatomical axis describes *where* and *how* disease is treated, including sites of cancer, treatment modalities, and patient-centered outcomes such as survival and toxicity. The mechanistic–molecular axis explains *how* cells respond to ionizing radiation, encompassing DNA damage and repair, gene-expression programs, and biomarker development.

These two axes intersect to form a translational continuum. Mechanistic and experimental advances feed into clinical decision-making. In turn, real clinical needs stimulate new waves of mechanistic research. Within this thematic space, several innovation fronts are advancing rapidly. Examples include ultrafast radiotherapy (FLASH-RT), immunoradiotherapy, biomarker-guided personalization, and predictive modeling.

Together, these fronts define a coherent and evolving ecosystem for precision radiotherapy. The co-occurrence supergraph (inset in Figure 2) provides a visual map of this ecosystem. In this graph, nodes correspond to keywords, edges represent co-occurrence frequency, and edge thickness and color indicate relationship strength. Thicker, reddish edges denote strong associations such as *cancer*, *radiotherapy*, *tumor*, and *particle*. Thinner, bluish edges represent weaker connections.

Structurally, the network exhibits high density and cohesion (density = 0.5, diameter ≈ 2, average degree = 9.5, weighted degree = 1962.2), indicating extensive interlinking throughout the literature. The combination of a high clustering coefficient (0.5) and very low modularity (0.02) shows that radiotherapy, radiobiology, and oncology no longer exist as isolated domains. They now merge into a single, tightly integrated thematic landscape.

Analysis of the undirected, thresholded version of the graph produced nearly identical node rankings (Pearson r > 0.9), confirming the robustness and internal consistency of the network topology. The low modularity indicates that thematic clusters are strongly interconnected rather than compartmentalized. The frequent co-occurrence of radiotherapy, radiobiology, and oncology terms reflects a mature scientific ecosystem in which molecular, physical, and clinical dimensions interact continuously. Mechanistic knowledge enables treatment personalization, while clinical demands motivate new modeling and experimentation.

The network’s high density confirms the shift from separate subfields to a unified translational domain. Within this integrated knowledge framework, biological discovery and clinical implementation operate together in a continuous feedback process.

LDA topic modeling applied to the same corpus revealed three dominant themes in the probabilistic distribution of words and articles. These correspond to the bidimensional structure of the field and explain how radiotherapy, radiobiology, and oncology became interconnected.

The first theme, translational, includes *radiation*, *effect*, *cell*, *combination*, *metastatic*, and *clinical* investigations that link preclinical or in vitro research with patient trials. The second, biomarker, features *cancer*, *trial*, *biomarkers*, *hypoxia*, and *radiosensitivity*, linking biological signatures and microenvironmental factors to therapeutic outcomes. The third, mechanistic, incorporates radiobiological modeling and machine learning to predict tissue responses under standard and ultrafast dose-rate conditions.

The co-occurrence supergraph and topic–term probability distributions show that these themes overlap as layers of a single semantic network. They demonstrate that molecular biology, medical physics, and clinical oncology now form one unified scientific ecosystem.

To validate this semantic structure, we refined searches across Scopus, PubMed, and Web of Science. A total of 61 representative publications explicitly connecting these three domains were retrieved. This curated corpus was used to verify the thematic hypotheses generated by the semantic models. The analysis confirmed that translational, biomarker, and mechanistic topics coexist and reinforce each other across the radiotherapy literature. Collectively, these findings demonstrate that radiotherapy, radiobiology, and oncology have evolved into a tightly interlinked interdisciplinary domain in which semantic cohesion supports true translational integration.

From this integration, four main hypotheses emerge:

1. *Cancer* and *radiotherapy* function as structuring axes of recent scientific production.
2. The growth of terms such as *cell*, *dose*, *effect*, and *treatment* reflects the emphasis on therapeutic personalization, mechanisms of action, and clinical efficacy.
3. The co-occurrence of *tumor*, *DNA*, *repair*, and *survival* indicates intensified research in precision medicine and response biomarkers.
4. The dense network connectivity confirms the transversal nature of the field, integrating molecular biology, medical physics, and clinical practice into a unified scientific ecology.

These hypotheses guided the search strategies used to locate research at the interface of radiotherapy, radiobiology, and oncology. In Scopus, we searched titles, abstracts, and author keywords restricted to biomedical subject areas for records published after 2014, retrieving 25 entries. In PubMed, we combined MeSH and free-text searches, limiting results to 2015–2024 and to clinical or translational publication types, yielding 10 records. In Web of Science, a similar query applied to abstracts returned 35 articles.

After automated deduplication by DOI and title, the records were consolidated into a corpus of 61 articles. This dataset centered on the molecular, therapeutic, and clinical trends of contemporary radiobiological oncology. It served to empirically validate the thematic and structural hypotheses identified through semantic analyses, providing a strong foundation for high-impact case study selection.

Semantic analysis of this corpus using LDA revealed a three-part thematic structure. When combined, topics extracted from titles collapsed into three primary themes.

The translational theme included *radiotherapy*, *effect*, *cell*, *combination*, *metastatic*, and *clinical*, reflecting studies that bridge therapeutic strategies in cellular systems with clinical trials. The biomarker theme included *cancer*, *patient*, *trial*, *biomarkers*, *hypoxia*, and *radiosensitivity*, corresponding to research linking molecular signatures and microenvironmental conditions with outcomes such as response and toxicity. The mechanistic theme included *model*, *tissue*, *flash*, *normal*, *genomics*, and *learning*, encompassing work that applies radiobiological modeling and machine learning to predict tissue responses, including those observed under ultrafast dose-rate conditions such as FLASH radiotherapy.

Together, these three themes outline a field that integrates laboratory findings, molecular profiling, and clinical practice while advancing data-driven approaches to predict and improve patient outcomes.

The analysis of abstracts confirmed this tripartite thematic structure while adding methodological refinement. Terms such as *response*, *damage*, *DNA*, *repair*, *radiosensitivity*, and *cellular* emphasized molecular responses to radiation. Words such as *parameter*, *model*, *radiobiological*, *high*, and *biological* reflected the development and calibration of quantitative models. Expressions such as *dose*, *risk*, *volume*, and *fractionation* indicated optimization of dosing schemes. The frequent presence of *signature*, *hypoxia*, *biomarker*, and *median cohort* revealed the adoption of genomic signatures and cohort analyses as predictive tools.

Co-occurrence analysis of the top 20 seed terms with the highest probabilities—*prediction*, *parameter*, *radiobiology*, *vitro*, *biology*, *personalized*, *protocol*, *effect*, *response*, and *cancer*—produced densely connected graphs. Terms such as *response*, *cancer*, *normal*, and *radiobiology* appeared as high-centrality bridge terms linking clinical and mechanistic themes. These terms connect outcomes, protocols, and therapeutic effects to cellular responses, radiobiological parameters, and in vitro findings.

In practice, these nodes function as semantic hinges that orient experimental modeling and biological insight toward clinical application. They unify previously separate strands of literature into a cohesive translational narrative.

To refine the selection of the most relevant articles for case studies, priority was given to expressions that combine molecular dimensions, clinical applications, and therapeutic innovation. Terms corresponding to the translational and mechanistic cores of the field—*biomarker signature*, *radiosensitivity*, *DNA repair*, *hypoxia-induced*, *FLASH radiotherapy*, *dose–response model*, *genomic classifier*, *precision oncology*, *radioresistance*, *therapeutic window*, *combined modality*, *clinical trial phase*, *translational framework*, *machine-learning prediction*, and *brachytherapy dose escalation*—were cross-referenced. This ensured that the literature analyzed remained closely aligned with the thematic axes generated by computational analysis and avoided dispersion into unrelated domains.

A total of 28 out of the 61 articles met these predefined criteria. Each included title or abstract contained at least one of the fifteen key expressions listed above. Articles without a DOI or those published outside the 2014–2025 window were excluded. Table 1 presents the selected articles, indicating publication year, identified terms, and the corresponding numerical classes assigned according to the established thematic axes [37,38,39,40,41,42,43,44,45,46,47,48,49,50,51,52,53,54,55,56,57,58,59,60,61,62].

Screening was conducted using a Python script that loaded the consolidated records, removed duplicates, and systematically examined the title and abstract fields. The algorithm implemented a lexical search function, converting text to lowercase and verifying term-by-term the exact presence of expressions derived from LDA modeling and co-occurrence patterns. For each record, lists of identified terms were generated and merged into a new column for structured thematic analysis.

The articles were then classified into five main classes: (1) DNA repair and molecular response, (2) precision oncology and genomic models, (3) individual radiosensitivity, (4) tumor radioresistance, and (5) emerging technologies in radiotherapy.

Each group was analyzed individually using the PaperProcessor [107] script, which performs semantic extraction and summarization guided by large language models (LLMs). The process was directed by the *subject* parameter, which guided thematic interpretation of each document. The parameter was adjusted according to the nature of each class to maintain analytical coherence and conceptual focus.

In the first four classes—focusing on molecular mechanisms, genomic stratification, clinical variability, and tumor resistance—open interpretative prompts generated explanatory syntheses, mechanistic inferences, and conceptual articulations. The fifth class, dedicated to technological innovation in areas such as FLASH radiotherapy, heavy charged particles, and voxel-level analytics, clarified physical principles, system design, and early clinical implementation. This structure enabled balanced and interpretative reading of the literature supported by artificial intelligence.

Although large language models are central to the semantic synthesis stage, their use within the PaperProcessor pipeline is tightly constrained. Independent preprocessing, normalization, and statistical validation steps precede all LLM analyses. This layered architecture ensures methodological transparency. All prompts are fixed and stored in the source code, allowing for full external auditing and exact reproducibility. Each model inference is time-stamped and logged in a CSV file to create a complete, auditable record of outputs.

An independent unsupervised topic modeling check using LDA is performed in parallel to verify consistency of themes across all articles. Together, these steps minimize interpretive bias and ensure that AI-generated synthesis remains evidence-based, transparent, and reproducible.

By analyzing these elements together, the study made the physical foundations of the techniques more tangible through models and quantitative metrics. The examination of device architectures and workflow constraints provided a realistic perspective on feasibility. Early clinical findings offered valuable reference points for assessing translational potential.

The discussion remained coherent and comparative, reflecting the main thematic axes of oncological radiotherapy in both its clinical and molecular dimensions. It also demonstrated how technological innovation serves as a connecting element that integrates these two domains within contemporary practice.

The questions used as subjects for each thematic class were as follows:

**Class 1—DNA Repair and Molecular Response:**

What are the key molecular responses to radiation discussed in the document, including DNA damage signaling, DNA repair pathways, and checkpoint activation mechanisms?

**Class 2—Precision Oncology and Genomic Modeling:**

How does the document address precision oncology, including the use of genomic profiling, machine learning models, and patient stratification in radiation therapy?

**Class 3—Individual Radiosensitivity and Clinical Risk:**

What evidence does the document present on interindividual radiosensitivity, clinical risk assessment, and predictive biomarkers for radiation response?

**Class 4—Radioresistance and Associated Mechanisms:**

What mechanisms of radioresistance are described in the document, including tumor hypoxia, metabolic reprogramming, stem cells, and viral integration?

**Class 5—Advanced Technologies and Innovative Radiotherapy:**

How does the document explore advanced radiotherapy strategies, including FLASH, hadron therapy, voxel-based analysis, and dose enhancement with high-Z nanoparticles?

Cosine similarity was used to measure the degree to which two texts align in feature space. For non-negative representations such as TF-IDF, the values range from 0 (no shared terms) to 1 (maximum lexical or semantic overlap).

For the 37 text pairs compared here, cosine similarity scores ranged from 0.291 to 0.669, spanning approximately 0.378. The overall mean was 0.5235 ± 0.0271 (95% CI: 0.4964–0.5506), indicating a moderate degree of similarity between the outputs of Llama3 (8B) and GPT-4o.

This moderate similarity was further analyzed by thematic class:

- **Class 1 (n = 6):** 0.4693 ± 0.1542 (0.3151–0.6235)
- **Class 2 (n = 6):** 0.5387 ± 0.0439 (0.4948–0.5826)
- **Class 3 (n = 18):** 0.5520 ± 0.0289 (0.5231–0.5810)
- **Class 4 (n = 4):** 0.4592 ± 0.0869 (0.3723–0.5460)
- **Class 5 (n = 3):** 0.5167 ± 0.2541 (0.2626–0.7708)

Classes 2 and 3 showed the highest means with the narrowest confidence intervals, suggesting that Llama3 (8B) reliably reproduces GPT-4o outputs in those domains. Classes 1 and 4 presented lower averages and wider intervals, reflecting greater variability and sensitivity to domain-specific terminology. Class 5 remained inconclusive due to its small sample size and wide uncertainty.

Cosine similarity served solely as a robustness check to compare lexical and semantic congruence between the two language models under identical prompts. It measured alignment but did not influence any bibliometric or clinical inference. The mean similarity of approximately 0.52 supports moderate cross-model reproducibility and methodological robustness. No hybrid inference or model fusion was performed. The outputs were compared only for completeness and terminological accuracy before manual curation. The notation “Llama → GPT-4o” indicates editorial workflow, where Llama3 generated the first draft and GPT-4o refined the language. This process improved clarity, consistency, and readability while maintaining all quantitative and bibliometric content.

Llama3 remains a valuable tool for fully offline analyses, which are essential for data security and sovereignty. The present comparison demonstrates cross-model consistency rather than hybrid modeling. It strengthens transparency and reproducibility in the analytical pipeline, maintaining the integrity of the results.

**Figure 3:**
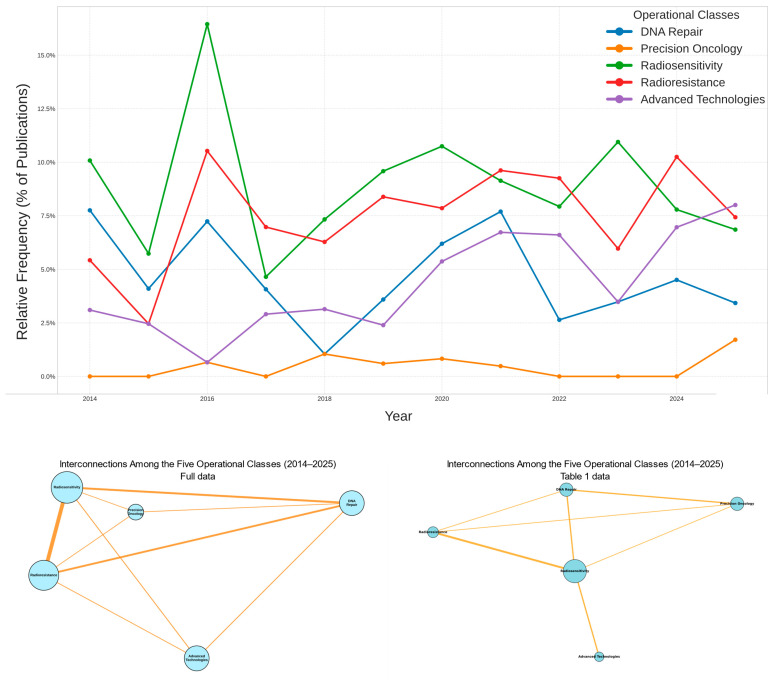
**Temporal and Structural Relationships Among the Five Operational Classes (2014–2025).** The top panel shows that Radiosensitivity and Radioresistance were the leading themes at the beginning of the era (2014) and reached their peak around 2016. Advanced Technologies maintained a steady presence throughout the decade, whereas Precision Oncology appeared only in the last few years. The bottom-left panel displays the co-occurrence network of all documents in the complete dataset, including the papers listed in Table 1. The central triad—Radiosensitivity, Radioresistance, and DNA Repair—represents the mechanistic core where cellular processes intersect with clinical outcomes. Advanced Technologies and Precision Oncology occupy more peripheral positions, marking the emerging frontiers of innovation. The bottom-right panel shows the reconstructed network for the 28 studies presented in Table 1. The central triad remains identical to that in the left panel, with Radiosensitivity as the principal node linking repair and response. Precision Oncology begins to bridge the mechanistic and clinical domains. Overall, the field is evolving from a research area centered on isolated mechanisms of response toward a comprehensive translational ecosystem. This shift marks a transition to a more integrated and interdisciplinary approach.

The data confirm the internal consistency of the analytical workflow and the robustness of model outputs across thematic domains. Figure 3 summarizes the evolving relationships and interdependencies among the five operational classes over time and through the network structure. It integrates molecular, clinical, and technological evidence into a unified representation of how precision radiotherapy is advancing and aligning with translational research.

Figure 3 illustrates the logic of the Integrated Implementation Plan for Precision Radiotherapy. It maps the five operational classes (blue) onto measurable health indicators (green) and broad public health goals (red).

In the top panel (2014–2024), Radiosensitivity remains the dominant theme for most of the period. Radioresistance follows a similar but steadier path. DNA Repair appears early and then declines. Advanced Technologies show continuous growth, becoming a dominant theme toward the end of the decade. Precision Oncology emerges later, reflecting the field’s shift toward genomics and personalized medicine.

The bottom-left panel presents the full-corpus co-occurrence network. Radiosensitivity and Radioresistance anchor the central hub, while DNA Repair functions as a bridging theme between mechanistic and clinical domains. Advanced Technologies and Precision Oncology occupy peripheral positions, indicating their ongoing integration into the radiobiological core.

The bottom-right panel, which represents the 28 studies in Table 1, recapitulates this structure at a smaller scale. Radiosensitivity again appears as the central hub. Precision Oncology now emerges as a bridging theme connecting molecular insight with clinical application.

Together, these findings confirm that the field is entering a stage where molecular and genomic evidence no longer remain descriptive but actively inform therapeutic decisions. Recent studies [98,99,100,101,102,103,142] show that molecular biomarkers and DNA repair biology increasingly guide clinical practice in precision radiotherapy. Genomic and transcriptomic profiles are now used to predict prognosis and treatment response in multiple tumor types, including prostate, breast, and head and neck cancers.

The elucidation of homologous recombination repair mechanisms and the clinical use of PARP inhibitors exemplify biology-driven approaches to radiosensitization. Liquid biopsies and circulating tumor DNA enable real-time monitoring of tumor evolution, supporting adaptive treatment strategies. Gene expression-based metrics of radiosensitivity, such as GARD, are being clinically tested to personalize dose delivery according to each patient’s genomic profile. These translational tools strengthen the connection between molecular insight and patient stratification, optimizing the precision, efficacy, and safety of radiotherapy.

AI-based evidence mapping is becoming increasingly powerful for guiding research funding, strengthening interdisciplinary capacity, and promoting equity in global radiotherapy. It enables stakeholders to identify thematic gaps, underrepresented regions, and emerging technological frontiers such as genomic radiosensitivity modeling, ultrafast dose-rate radiotherapy, and adaptive treatment planning. These insights support evidence-based decisions that maximize translational impact.

The Intelligent Cancer Catchment Area Tool (iCAT) exemplifies how geographic information can be combined with machine learning to identify high-risk, under-studied, and under-resourced regions [143,144,145,146,147,148,149,150,151,152]. Bibliometric mapping can also reveal mismatches between disease burden and research activity, particularly in low-resource settings [144,147]. This information helps funding agencies redirect investments to areas where scientific progress can most effectively reduce inequities [151].

AI-based mapping further identifies institutional and geographic clustering, informing the creation of targeted multidisciplinary training programs. Such programs can bridge computational oncology, radiobiology, and public health analytics, building a workforce capable of translating molecular and data-driven insights into clinical benefit [146].

AI-enhanced radiotherapy is already demonstrating its impact on equity in precision oncology. It improves access, personalization, and outcomes across diverse populations. Virtual and augmented reality technologies can extend care and education to underserved communities, reducing geographic and socioeconomic barriers [148].

For these systems to achieve fair and lasting global impact, they must be deployed ethically. Attention to bias, privacy, and data governance is essential to ensure that AI-driven progress in radiotherapy advances equity as well as innovation.

### 3.4. Operational Pathways for Dose Personalization and AI-Driven Adaptation

Translating biomarker discoveries into meaningful clinical benefit in precision radiotherapy requires converting molecular findings into actionable tools for dose personalization and treatment adaptation as patients evolve over time. In recent years, this vision has developed into three closely interrelated approaches: genomic dose personalization, voxel-level dose painting, and AI-driven adaptive radiotherapy.

Together, these strategies represent a paradigm shift from population-based prescriptions to biologically informed, patient-specific planning. This transition marks a crucial step toward incorporating radiobiological understanding into the daily decision-making processes of clinical practice.

#### 3.4.1. Genomic Dose Personalization (RSI/GARD)

In this context, genomic radiosensitivity indices (RSI) and the genomic-adjusted radiation dose (GARD) have become widely recognized as practical and measurable strategies for personalizing radiotherapy according to the tumor’s intrinsic biology. These concepts were originally described and validated by Torres-Roca and colleagues, and later expanded in clinical contexts by Scott et al. (2021) [153].

Both approaches convert gene expression signatures into predictors of radiation response. This enables clinicians to modulate the radiation dose so that the intended biological effect more closely aligns with therapeutic goals. Recent work has focused on translating RSI and GARD from bench to bedside, evaluating their use as personalized radiation therapy tools designed to improve efficacy and patient outcomes through dose and fractionation adjustments based on the tumor’s genomic profile.

In triple-negative breast cancer (TNBC), Stecklein et al. (2024) [154] found that RSI–GARD scores remain consistent before and after neoadjuvant systemic therapy. The score reflects how well a tumor responds to treatment and can guide clinicians in deciding whether to increase or decrease radiation doses based on genetic profiles. A Phase II clinical trial is also investigating whether customizing radiation doses according to genomic information can improve outcomes in breast-conserving therapy, with a primary goal of improving local tumor control [155].

In rectal cancer, Xia et al. (2024) [156] confirmed that the GARD-based model can classify patients by prognosis, with higher GARD scores associated with better clinical outcomes. Yuan et al. (2020) [157] reported that RSI-guided dose escalation may increase pathologic complete response rates. In the adjuvant breast setting, Ahmed et al. (2016) [158] showed that GARD significantly predicts local recurrence, identifying a subgroup of patients who may benefit from selective dose intensification.

The predictive utility of GARD extends beyond breast and rectal cancer. In head and neck malignancies, Ho et al. (2025) [159] demonstrated that GARD outperforms traditional clinical predictors in HPV-positive oropharyngeal cancer, supporting its potential role in guiding safe dose de-escalation. In nasopharyngeal carcinoma, Chiang et al. (2024) [160] used GARD to individualize dose plans aimed at enhancing locoregional control and reducing treatment failure.

In glioblastoma, Ahmed et al. (2015) [161] found that GARD predicts overall survival in patients with high MGMT expression, suggesting that biologically guided dose escalation may benefit more resistant subgroups. A pan-cancer analysis by Scott et al. (2017, 2021) [153,162] further underscored the broad relevance of GARD, linking higher scores to improved recurrence-free and overall survival across numerous tumor types [153,154,155,156,157,158,159,160,161,162].

Recent clinical evidence confirms that genomic biomarkers such as RSI and GARD are being operationalized in Phase II trials to guide fractionation and dose prescriptions based on individual tumor radiosensitivity. Validation across different cancer types—including breast, rectal, and HPV-positive oropharyngeal cancers—shows that applying RSI–GARD models in clinical practice facilitates dosage adjustments informed by physiological data, resulting in measurable therapeutic benefits [159,163,164,165,166,167,168,169,170,171].

In summary, these investigations highlight the transformative potential of RSI and GARD for radiotherapy personalization. They also emphasize the practical challenges of integrating these tools into clinical workflows, standardizing assays, and ensuring cost-effectiveness. Widespread adoption will require harmonized bioinformatics pipelines, multicenter validation, and incorporation of these metrics into clinical decision-support systems.

Nonetheless, RSI and GARD represent a critical step toward biologically calibrated radiation dosing—replacing empirical, fixed-dose strategies. They mark the emergence of a precision oncology paradigm in which genomic data directly guide clinical intervention.

#### 3.4.2. Voxel-Level Dose Painting

Despite the progress of whole-tumor genomics, intratumoral heterogeneity remains a major barrier to consistent treatment outcomes. Voxel-based dose painting offers a practical path forward by redistributing the radiation dose according to the tumor’s spatial biology, as reflected in molecular, functional, or radiomic biomarkers.

This approach uses multimodal imaging to identify resistant niches, such as hypoxic or highly metabolically active subvolumes. It then selectively escalates the dose to these targets while sparing normal tissues. This enables precise targeting of regions that require more intensive treatment.

Li et al. (2025) [172] demonstrated that hypoxia-guided dose painting in lung cancer is both promising and feasible. Incorporating 18F-FMISO PET-CT into treatment planning increased the likelihood of tumor control by approximately 24.5% and reduced side effects in healthy tissues by 1.8% compared with standard plans. These findings show that biologically informed dose shaping can provide meaningful clinical benefits.

In head and neck cancer, Yan et al. (2019) [173] developed an adaptive dose-painting method using FDG-PET/CT scans to generate real-time voxel-level maps of tumor response. This technology identifies resistant tumor areas during treatment and modifies the dose dynamically, enabling therapy to evolve with the tumor.

Radiomics expands the potential of biologically adaptive radiotherapy by providing a detailed characterization of tumor features. The Rad-TRaP framework (Shiradkar et al., 2016 [174]) employs multiparametric MRI to identify specific prostate cancer lesions and generate personalized treatment plans. This approach protects surrounding organs while ensuring effective tumor control.

Thorwarth (2018) [175] advanced biologically adaptive radiotherapy by linking dose delivery to the tumor’s evolving functional imaging profile. This real-time adaptation aligns treatment with biological changes observed during therapy. Early translational studies already support the move toward clinical implementation.

Almeldin et al. (2023) [176] demonstrated the feasibility of Biological Image-Guided Adaptive Radiotherapy (BIGART) in glioblastoma. Using advanced MRI techniques, they pinpointed resistant tumor regions and selectively increased radiation dose while sparing healthy tissue. Similarly, Naghavi et al. (2024) [177] introduced Habitat Escalated Adaptive Therapy (HEAT), which integrates radiomic habitats with GARD-based genomic optimization in soft tissue sarcoma. This approach improved pathological response rates and set a precedent for combined biological planning.

Recent studies in voxel-based radiotherapy have established clinically applicable protocols in which biomarker-positive subvolumes, identified by molecular imaging or genomic profiling, receive planned voxel-level dose escalations. These boosts are designed within normal tissue complication probability (NTCP) limits to ensure safety while improving tumor control.

In practical terms, this corresponds to a simple biological dose-painting rule: increase the dose by approximately 10–15% in biomarker-positive regions, provided that NTCP constraints are met. This allows targeted intensification without exceeding normal tissue tolerance [159,178,179,180,181,182,183,184,185,186].

Despite its technical maturity, the successful implementation of voxel-based approaches depends on standardized imaging protocols, robust and reproducible biomarker quantification methods, and validation in large prospective cohorts. These components are essential to ensure treatment accuracy and reliability.

Collectively, these studies demonstrate that tumor biological heterogeneity can be translated into spatially resolved treatment strategies. Dose painting thus emerges as a practical embodiment of precision medicine, in which radiation delivery is tailored to the molecular and functional landscape of each tumor. This approach has significant potential to enhance treatment outcomes [172,173,174,175,176,177,187,188,189,190].

#### 3.4.3. AI-Driven Adaptive Radiotherapy

The combination of artificial intelligence (AI) and long-term biomarkers has enabled a new paradigm for adaptive radiotherapy based on *measure–predict–adapt* cycles. These cycles integrate real-time imaging, biological monitoring, and algorithmic prediction to adjust treatment as tumors evolve during therapy.

A key component of this strategy is the integration of liquid biopsy dynamics, particularly circulating tumor DNA (ctDNA), with AI-based planning. ctDNA reflects tumor burden and clonal evolution in real time, and its on-treatment kinetics may indicate therapeutic response before anatomical imaging becomes informative. Janke et al. (2025) [191] demonstrated that lower ctDNA levels during re-irradiation are associated with improved outcomes, whereas higher levels correlate with early relapse.

When ctDNA dynamics are combined with radiomic features derived from FDG-PET, predictive modeling for risk stratification and adaptive planning improves markedly in non-small cell lung cancer [192,193]. Collectively, these studies support the use of molecular dynamics as quantitative triggers for dose adaptation within a closed feedback loop.

AI is also accelerating anatomical adaptation, particularly through AI-based adaptive radiotherapy (AI-ART) systems. These systems use daily cone-beam CT (CBCT) to automatically segment targets and organs at risk, reoptimize dose distributions, and generate updated plans within minutes. They are especially valuable in head and neck cancers, where anatomical changes are frequent during treatment.

Studies by Salhab et al. (2023) [194], Sher et al. (2023) [195], and Blumenfeld et al. (2022) [196] have shown that AI-based ART improves target coverage and enhances sparing of critical structures, thereby increasing the therapeutic index. Deep learning methods have also accelerated contouring and replanning, enabling true online adaptation [197,198].

AI-based adaptive radiotherapy and longitudinal biomarkers such as ctDNA are now approaching clinical translation. These tools offer the potential for real-time treatment re-optimization guided by biological feedback rather than static planning. Although widespread clinical implementation remains under development, existing evidence indicates that adaptive frameworks can modify treatment delivery in response to evolving biomarker signals.

In practice, this enables a *measure–predict–adapt* cycle. A sustained decline in ctDNA would support continued therapy or even de-escalation. Stable or rising ctDNA levels would prompt replanning, including focal dose escalation to resistant subregions. This approach represents the next logical step in the evolution of personalized radiotherapy. AI-driven adaptation and longitudinal biomarker measurement bring dose delivery into closer alignment with tumor biology, enhancing precision and improving outcomes [146,159,163,179,193,199,200,201,202,203].

Future adaptive radiotherapy will fuse multimodal data—including ctDNA kinetics, radiomic phenotypes, and dosimetric feedback—into predictive AI models capable of prompting mid-course re-optimization. These frameworks operationalize biological adaptation by linking dose modulation directly to tumor behavior [191,192,193,194,195,196,197,198,204,205].

In summary, the future of adaptive radiotherapy lies in integrating multiple data streams—ctDNA trajectories, radiomic features, and real-time dose information—into AI systems that predict when and how to modify treatment mid-course. Such systems make biological adaptation achievable by correlating dose changes with tumor dynamics during therapy.

Significant challenges remain, including standardization of biomarker assays, model transparency, and the demonstration of clinical utility in prospective trials. However, early evidence supports the feasibility of continuously learning, response-driven systems. Radiotherapy is evolving from fixed treatment plans toward adaptive intelligence. This comprehensive approach aims to deliver the right dose to the right target at the right time while uniting molecular insight with clinical action, as highlighted by Balázs et al. (2024) [204].

### 3.5. Case Studies

Among the studies automatically selected and classified by our semantic model (detailed in Table 1), representative examples for each thematic class are summarized below. These examples were derived directly from the LDA-based classification and reflect the evidence retrieved by the computational pipeline rather than manually curated selections.

**Class 1—DNA Repair and Molecular Response.**

These studies focus on the molecular mechanisms underlying radiation response, including DNA repair, checkpoint regulation, and therapeutic modulation.

Norbert Mészáros et al. (2019) [138] reported progressive breast fibrosis in patients with extreme radiosensitivity. This phenotype was associated with high rates of chromosomal aberrations, supporting the use of cytogenetic assays for risk stratification.

Luis Bermúdez-Guzmán et al. (2021) [116] demonstrated that chronoradiotherapy—by aligning dose delivery with the circadian rhythms of genes such as *BMAL1*, *CLOCK*, *PER*, and *CRY*—accelerates DNA break resolution and reduces toxicity.

Michael D. Story et al. (2024) [115] showed that high-LET radiation induces complex DNA lesions and activates the cGAS–STING pathway. Radiosensitization was enhanced by inhibition of HR, NHEJ, ATM, and ATR.

Olga A. Martin et al. (2024) [117] reviewed 75 years of radiobiology, highlighting the influence of radiation quality on γH2AX persistence and the modulation of the tumor microenvironment.

Davide Perico and Pierluigi Mauri (2025) [141] correlated overexpression of RAD51 and BRCA1, as well as hyperactivation of NHEJ, with radioresistance.

Yan Luo (2025) [118] demonstrated that high-Z nanoparticles amplify DNA damage and reactive species generation, proposing standardized evaluation metrics such as NER and SER.

**Class 2—Precision Oncology and Genomic Modeling.**

Ivana Dokic et al. (2016) [120] used ion beams and the Cell-Fit-HD technology to show that persistence of γH2AX foci at 72 h is a stronger biomarker of radiosensitivity than the initial lesion count. This finding supports the development of biodosimetric repositories.

Paolo Tini et al. (2018) [122], analyzing 17,412 cases of lung carcinoma, showed that radiotherapy provides the greatest benefit in advanced stages and squamous histology, underscoring the value of integrating molecular data into treatment planning.

Henning Willers et al. (2019) [123] proposed the use of RSI and GARD to guide personalized dosing.

Perico and Mauri (2025) [141] identified proteins such as RAD51, PARP1, CHK1, and MAPK15 as mediators of resistance.

Marco Calvaruso et al. (2025) [121] argued for the integration of multi-omic biomarkers and artificial intelligence to improve prediction of treatment response.

Yan Luo (2025) [118] developed a multidimensional index for high-Z nanoparticles that incorporates biological variability and immune modulation.

The most important biomarkers associated with tumor response to therapy (progression versus regression) are listed in Table 2. The table is organized by biomarker type: molecular, blood-based, proteomic, immune, and imaging biomarkers.

In addition, multi-omic results from diverse clinical contexts are summarized. Mechanisms, predictive accuracy, and limitations for each biomarker type are discussed. The goal of the table is to provide a clear, clinically oriented summary of the key biomarkers relevant to precision radiotherapy and to identify where the supporting evidence is strongest or still evolving.

**Class 3—Individual Radiosensitivity and Clinical Risk**

This class gathered the largest number of studies. Igor Sirák et al. (2015) [132] reported *FANCA* mutations associated with severe toxicities. Adeline Granzotto et al. (2016) [140] identified ATM nucleoshuttling kinetics as a functional biomarker of radiosensitivity. Yasuhiro Ogawa (2016) [133] introduced KORTUC II, a hydrogen peroxide–based radiosensitizer.

Sarah L. Kerns et al. (2018) [134] reviewed the application of RSI and GARD, while E.J. Her et al. (2018) [135] modeled tumor control probability (TCP) for prostate brachytherapy. Nathalie Arians et al. (2019) [125] demonstrated that carbon ions can overcome HPV-induced resistance. Mészáros et al. (2019) [138] correlated cytogenetic profiles with the development of severe fibrosis.

John Kang et al. (2020) [131] reviewed genomic and machine learning models for radiosensitivity prediction. Chekhun and Domina (2021) [128] suggested that COVID-19 infection may increase individual radiosensitivity. Dayyani et al. (2021) [139] compared ^60^Co and ^192^Ir in cervical brachytherapy, while Cesare Cozzarini et al. (2022) [126] developed a TCP model validated in 795 post-prostatectomy patients.

Nikitović and Stanojković (2022) [130] linked microRNAs and cytokines to prostate cancer toxicity. Verduijn et al. (2022) [136] developed the COMPLETE protocol, integrating multiparametric imaging, omics data, and machine learning. Raitanen et al. (2023) [129] demonstrated greater radioresistance in 3D spheroids. McWilliam et al. (2023) [137] applied voxel-based analysis to map critical anatomical regions associated with toxicity.

Jazmati et al. (2025) [127] investigated pediatric medulloblastoma, identifying homogeneous α/β ratios. Perico and Mauri (2025) [141] emphasized the role of proteomics in identifying radioresistance profiles. Guo et al. (2025) [15] reviewed the mechanisms of FLASH-RT, including oxygen depletion and mitochondrial preservation, highlighting its integration with immunotherapy.

**Class 4—Radioresistance and Associated Mechanisms**

Granzotto et al. (2016) [140] linked delayed ATM nucleoshuttling to radioresistance. Mery et al. (2018) [124] demonstrated that VHL mutations induce pseudohypoxia in renal carcinoma through HIF activation, suggesting that carbon ions and repair inhibitors represent viable therapeutic options.

Arians et al. (2019) [125] confirmed that carbon ions restore checkpoint control in HPV-positive cervical tumors with E2 disruption, with relative biological effectiveness (RBE) values ranging from 1.3 to 4.3. Perico and Mauri (2025) [141] integrated pathways of hypoxia, metabolism, stemness, viral integration, and apoptosis evasion into comprehensive proteomic maps. They advocated the use of these maps for biomarker discovery and selective therapy design.

**Class 5—Advanced Technologies and Innovative Radiotherapy**

This class focuses on the adoption of cutting-edge modalities in precision treatment. Zhang et al. (2021) [119] investigated FLASH radiotherapy (≥40 Gy/s), demonstrating comparable tumor control to conventional RT but with superior tissue preservation and immune modulation. Effects included lymphocyte sparing and tumor remodeling with enhanced CD8^+^ infiltration.

Dayyani et al. (2021) [139] compared ^60^Co and ^192^Ir for cervical cancer brachytherapy. Both isotopes were shown to be effective with appropriate dose adjustments. ^60^Co presented physical advantages, while ^192^Ir exhibited a higher RBE.

Guo et al. (2025) [15] further explored FLASH-RT mechanisms, including free radical modulation, mitochondrial preservation, and vascular integrity maintenance. They emphasized the importance of dosimetric standardization and voxel-based mapping for clinical translation. Collectively, these studies consolidate FLASH-RT and optimized brachytherapy as key components of next-generation personalized treatment protocols.

The synthesis of these case studies forms the scientific foundation of the Integrated Implementation Plan for Precision Radiotherapy. Each thematic class—from molecular DNA response to advanced delivery technologies such as FLASH-RT—reveals mechanisms, biomarkers, models, and strategies that can be directly translated into personalized clinical protocols.

By organizing these findings into five interdependent operational blocks, the plan transforms experimental and translational evidence into a continuous flow of investigation, validation, and clinical application. This framework supports both individualized therapeutic progress and the integration of objective indicators into public health policy, establishing a structured pathway for the advancement of precision radiotherapy.

### 3.6. Implementation Plan in Precision Radiotherapy

The Integrated Implementation Plan in Precision Radiotherapy (IIPPR) is organized into five classes that establish a continuous flow of investigation, validation, and clinical application. This structure also allows for direct integration into public health policies.

**Class 1—DNA Repair and Molecular Response** constitutes the operational block that provides the molecular and functional foundation for all subsequent stages. Its objective is to identify DNA repair mechanisms, regulate checkpoints, and characterize signaling pathways that determine each patient’s response to radiation. This step yields essential biomarkers and functional parameters for therapeutic personalization. It enables biologically precise decisions regarding technique, dose, fractionation, and adjuvant combinations from the beginning of the workflow. Implementation requires functional assays, omics analyses, and molecular modeling at multiple levels of complexity.

Norbert Mészáros et al. (2019) [138] reported that patients with progressive radiation-induced breast fibrosis exhibited high frequencies of chromosomal aberrations, even in non-irradiated cells. This finding demonstrated that genomic instability and persistent checkpoint failure can be detected cytogenetically and used as predictive indicators.

Luis Bermúdez-Guzmán et al. (2021) [116] showed that synchronizing irradiation with circadian phases of maximal repair activity accelerates the resolution of double-strand breaks. This temporal modulation improves the efficiency of HR, NHEJ, NER, and BER, reducing toxicity and enhancing efficacy.

Michael D. Story et al. (2024) [115] demonstrated that high-LET radiation, such as that used in hadron therapy, induces complex lesions and activates immune pathways via cGAS–STING. Radiosensitization can be further amplified by pharmacological inhibition of HR, NHEJ, ATM, and ATR.

The review by Olga A. Martin et al. (2024) [117] reinforced the role of radiation quality in γH2AX persistence, tumor microenvironment remodeling, and integration with immunotherapy.

Davide Perico and Pierluigi Mauri (2025) [141] highlighted proteomic integration as a strategy to identify functional profiles of key proteins associated with radioresistance. Yan Luo (2025) [118] demonstrated that high-Z nanoparticles enhance radiosensitization by overloading DNA repair pathways and proposed standardization of response metrics such as NER and SER.

The central action of this class is the integration of molecular and functional information into a validated panel of predictive biomarkers. This panel provides subsequent phases with a comprehensive map of tumor vulnerabilities and the repair limitations of normal tissues.

**Class 2—Precision Oncology and Genomic Modeling** transforms molecular and radiobiological data into actionable parameters that guide the selection of technique, dose, and fractionation. It replaces empirical protocols with biologically informed prescriptions.

Ivana Dokic et al. (2016) [120] used Cell-Fit-HD technology to show that repair kinetics is a more reliable biomarker than the initial lesion count. The authors proposed the creation of biodosimetric repositories to support biological dose prescriptions.

In a population-based analysis of 17,412 cases of non-small cell lung carcinoma, Paolo Tini et al. (2018) [122] demonstrated the importance of integrating genomic markers and predictive models to improve patient selection.

Henning Willers et al. (2019) [123] introduced indices such as RSI and GARD to calibrate dose based on gene expression, dosimetry, and imaging. These indices enable integration with immunotherapy and real-time treatment adjustment.

Davide Perico and Pierluigi Mauri (2025) [141] incorporated functional proteomic layers into patient stratification. They identified RAD51, PARP1, CHK1, and MAPK15 as key proteins whose integration with transcriptomic data enhances predictive power.

Marco Calvaruso et al. (2025) [121] demonstrated that multi-omic biomarkers, when combined with FLASH-RT and predictive algorithms, allow for fine-tuning of intensity and technique while minimizing toxicity.

Yan Luo (2025) [118] developed a multidimensional index integrating biological variability, immune modulation, and quantitative radiobiological metrics, including DER, SER, and RBE.

Recent research in endometrial cancer underscores the importance of genomic modeling for radiotherapy optimization through molecular classification. The PORTEC-3 study distinguished POLE-mutated from TP53-mutated tumors, showing that this classification predicts both treatment response and prognosis. This finding supports adaptive dosing based on molecular phenotype. The ongoing PORTEC-4 trial expands this approach by incorporating molecular data directly into therapeutic decision-making to achieve biologically guided radiotherapy optimization.

The TCGA classification identifies four subtypes—POLE-mutated, MMRd, p53abn, and NSMP—each with distinct prognostic significance. POLE and MMRd tumors are associated with favorable outcomes, while p53abn tumors correspond to poor prognosis [223,224,225,226,227,228,229,230,231,232].

Incorporating these molecular markers into clinical workflows enables precise and personalized radiotherapy that improves both efficacy and patient outcomes. This class delivers a therapeutic plan calibrated to the individual tumor and patient, validated in predictive simulations, and ready for clinical application.

**Class 3—Individual Radiosensitivity and Clinical Risk**

This class focuses on identifying and integrating the variables that define individual tolerance to radiation. It adjusts therapeutic protocols according to genetic, functional, and clinical predispositions.

Igor Sirák et al. (2015) [132] documented exacerbated toxicity in a patient with *FANCA* heterozygosity, while Adeline Granzotto et al. (2016) [140] demonstrated that delayed nuclear translocation of ATM compromises DNA repair. Both studies provide examples of biomarkers suitable for pre-treatment screening.

Yasuhiro Ogawa (2016) [133] introduced the KORTUC II method, which employs hydrogen peroxide modulation to enhance radiosensitivity. Sarah L. Kerns et al. (2018) [134] and John Kang et al. (2020) [131] explored the clinical relevance of RSI and GARD in association with TCP and NTCP models. E.J. Her et al. (2018) [135] highlighted the influence of α-parameter variability in prostate brachytherapy.

Nathalie Arians et al. (2019) [125] showed that carbon ions can overcome HPV-induced resistance, while Norbert Mészáros et al. (2019) [138] reinforced the predictive value of cytogenetic assays. V.F. Chekhun and E.A. Domina (2021) [128] extended the discussion to systemic factors, including those associated with COVID-19.

Mahdieh Dayyani et al. (2021) [139] compared ^60^Co and ^192^Ir sources in brachytherapy. Cesare Cozzarini et al. (2022) [126] developed TCP models validated in post-prostatectomy cohorts. Marina Nikitović and Tatjana Stanojković (2022) [130] compiled molecular and clinical evidence linking biomarkers to toxicity outcomes. Gerda M. Verduijn et al. (2022) [136] consolidated the COMPLETE protocol, integrating multiparametric imaging, radiomics, and machine learning.

Recent studies have expanded this translational foundation. Raitanen et al. (2023) [129] employed 3D spheroids to model cellular responses. McWilliam et al. (2023) [137] applied voxel-based mapping to identify critical regions of radiosensitivity. Jazmati et al. (2025) [127] contributed data from pediatric radiobiology, while Perico and Mauri (2025) [141] emphasized functional proteomics in the identification of radioresistance profiles. Guo et al. (2025) [15] examined FLASH-RT mechanisms involving oxygen depletion and mitochondrial preservation.

The central objective of this class is to consolidate an individual risk matrix that combines molecular biomarkers and clinical parameters. This matrix translates into operational recommendations for dose, technique, and modality selection tailored to each patient.

**Class 4—Radioresistance and Associated Mechanisms**

This class focuses on identifying and counteracting tumor mechanisms that confer resistance to radiation.

Adeline Granzotto et al. (2016) [140] showed that delayed ATM phosphorylation kinetics compromise target activation. Mery et al. (2018) [124] demonstrated that VHL mutations in renal carcinoma trigger pseudohypoxia through HIF activation, promoting pro-survival signaling.

Nathalie Arians et al. (2019) [125] found that in HPV-positive cervical cancer, carbon-ion irradiation restores checkpoint control by reversing p53 and Rb degradation. Davide Perico and Pierluigi Mauri (2025) [141] expanded this framework using high-resolution proteomics. They integrated molecular pathways of hypoxia, metabolism, stemness, viral integration, and apoptosis evasion into detailed proteomic maps.

This class translates such molecular mapping into targeted interventions. Strategies include high-LET modalities, repair inhibitors, and microenvironment modulators aimed at reversing resistant phenotypes and improving therapeutic outcomes in poor-prognosis subgroups.

**Class 5—Advanced Technologies and Innovative Radiotherapy**

This class addresses the integration of next-generation radiotherapy modalities into clinical workflows.

Zhang et al. (2021) [119] demonstrated that ultrafast radiotherapy (FLASH-RT, ≥40 Gy/s) maintains tumoricidal efficacy while preserving normal tissue integrity and remodeling the immune microenvironment. Guo et al. (2025) [15] confirmed the clinical feasibility of FLASH-RT and identified multiple protective mechanisms, including mitochondrial preservation and CD8^+^ lymphocyte maintenance, provided it is supported by dosimetric standardization.

Mahdieh Dayyani et al. (2021) [139] compared ^60^Co and ^192^Ir sources in cervical brachytherapy, demonstrating that dose optimization can balance biological effectiveness and safety. Despite their established status, both isotopes remain in this class due to ongoing technological improvements in dosimetric modeling, delivery precision, and biological weighting, which continue to advance brachytherapy techniques for next-generation applications.

This class integrates FLASH-RT, optimized brachytherapy, and voxel-based mapping into adaptive protocols that unite physical precision with biological selectivity.

#### Synthesis and Implementation Framework

Together, these five classes outline a pragmatic framework that connects **precision** radiotherapy to population-level healthcare delivery. For Brazil’s Unified Health System (SUS), the plan defines how standardized workflows—such as molecular diagnostics, biomarker-driven patient stratification, and adaptive radiotherapy protocols—can be implemented progressively, contingent upon clinical validation.

Rather than suggesting immediate outcomes, the framework emphasizes the interfaces between biomarker discovery, dosimetric modeling, and outcome assessment. Once validated in pilot environments, these interfaces will enable evidence-based decision-making and the rational allocation of limited healthcare resources.

Table 3 organizes prospective metrics by class, describing how indicators such as survival, toxicity, personalization, access, and cost-effectiveness can be measured in pilot or registry-based studies.

**Figure 4:**
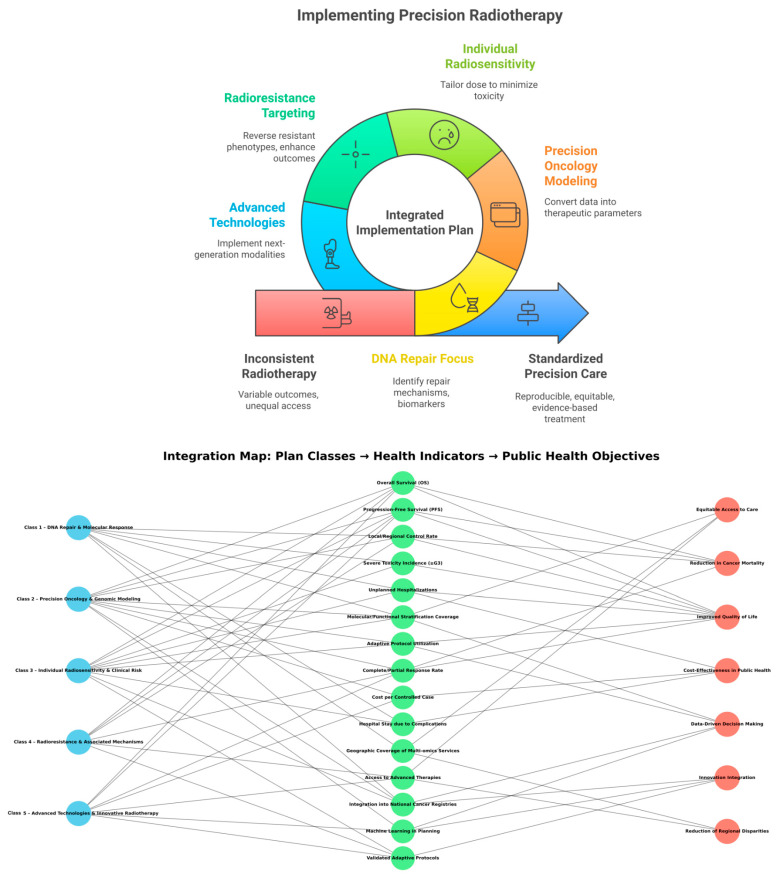
**Integration between Plan Classes, Health Indicators, and Strategic Objectives.** The figure illustrates how the five classes of the Integrated Implementation Plan in Precision Radiotherapy (IIPPR) connect across three levels. Blue nodes (left) represent the operational classes. Green nodes (center) correspond to measurable health indicators. Red nodes (right) denote broader public health objectives. The network visualizes the translation pathway from technical and scientific actions to clinical and societal outcomes. These actions include DNA repair characterization, genomic and proteomic modeling, individualized radiosensitivity assessment, targeting of radioresistance mechanisms, and the adoption of advanced radiotherapy technologies. Together, these processes generate quantifiable outputs such as overall survival (OS), progression-free survival (PFS), local and systemic control, incidence of severe toxicity (G ≥ 3), and cost per controlled case. These indicators converge toward collective health objectives within the Brazilian Unified Health System (SUS). They support equitable access to care, reduced cancer mortality, and improved quality of life. They also reinforce cost-effectiveness in oncology, data-driven decision-making, technological innovation, and the reduction in regional disparities.

Figure 4 presents a conceptual integration map that connects the plan’s operational classes (blue nodes), measurable indicators (green nodes), and strategic health objectives (red nodes). The figure illustrates a logical progression from scientific discovery to policy evaluation, without implying causality.

This structure positions the IIPPR as a dynamic and testable framework that links molecular biology, clinical radiotherapy, and health management in a coherent model. It remains open to validation through prospective cohorts and pilot programs within the SUS.

The Implementation Plan should not be viewed as a rigid timeline, but as a living and adaptable roadmap for development. Its pace must remain coordinated through three interdependent components moving in lockstep: (i) scientific and clinical validation, (ii) regulatory readiness, and (iii) public sector engagement. This alignment ensures the plan can adjust dynamically to changing circumstances.

The plan evolves through three maturity levels.

(i) **Scientific and clinical validation** focuses on biomarker qualification and the harmonization of data standards across institutions.
(ii) **The translational phase** emphasizes interoperability, real-world pilot programs within national health systems, and the establishment of governance standards for AI-assisted radiotherapy.
(iii) **The consolidation phase** centers on the integration of adaptive, biomarker-driven protocols and AI ethics frameworks into public policy and clinical guidelines.

Progression from one maturity level to the next occurs through collaborative coordination and negotiated consensus among research institutions, regulatory agencies, and funding bodies.

Your role is essential in guiding the development of precision radiotherapy so that it evolves in alignment with national priorities, equity objectives, and technological capacity.

## 4. Discussion

### 4.1. Overview and Conceptual Integration

This paper presents a timely, concise, and human-centered account of how radiotherapy, radiobiology, and oncology have evolved—and continue to evolve—based on the AI-driven semantic and temporal analysis of 3343 publications published between 1964 and 2025.

The principal strength of this study lies in its methodological integration. It combines rigorous data cleaning and normalization (including deduplication and lemmatization) with advanced discovery tools such as topic modeling and network analysis. Temporal mapping further reconstructs the conceptual architecture of the field, revealing two complementary axes that define its current structure.

The clinical–anatomical axis organizes disease sites, therapeutic modalities, and prognostic endpoints. In contrast, the mechanistic–molecular axis encompasses DNA damage and repair, biomarker identification, and cellular radiation responses. Together, these axes describe a field that has evolved from isolated domains into an interdependent system, where biological mechanisms and clinical decision-making inform each other in near real time.

### 4.2. Translational Framework: The Precision Radiotherapy Implementation Plan (PRIP)

#### 4.2.1. Structure and Thematic Domains

The most practical outcome of this work is the Precision Radiotherapy Implementation Plan (PRIP). This framework operationalizes the integrated understanding of the field into five distinct classes: (i) DNA repair and molecular response, (ii) precision oncology and genomic modeling, (iii) individual radiosensitivity and clinical risk, (iv) mechanisms of radioresistance, and (v) innovative radiotherapy technologies. Each class integrates biomarkers, predictive models, and therapeutic strategies to support individualized care.

#### 4.2.2. Translational Role and Health-System Relevance

Importantly, this framework is not conceptual but translational. It bridges data and clinical application by aligning AI-derived biological markers with real-world outcomes that matter to both patients and healthcare systems: overall survival (OS), progression-free survival (PFS), toxicity profile, and cost per controlled case. In a scalability- and equity-oriented environment such as Brazil’s Unified Health System (SUS), the plan provides a pathway to integrate molecular diagnostics and predictive stratification into standardized, resource-sensitive workflows.

It also outlines a feasible strategy for the responsible implementation of advanced modalities, such as FLASH radiotherapy and hadron therapy, by linking their adoption to measurable clinical benefits and demonstrated feasibility.

### 4.3. Clinical and Operational Implications

#### 4.3.1. Predictive and Adaptive Applications

Clinically, the proposed framework supports predictive and adaptive radiotherapy, enabling patient stratification based on DNA repair kinetics, intrinsic radiosensitivity, and radioresistance pathways. Such integration can drive truly personalized treatment plans. When implemented prospectively within hospital systems—linked to EHRs, PACS, molecular diagnostics, and adaptive planning software—AI-driven semantic models could function within standard clinical workflows. This would enable more accurate treatments, reduced toxicity, and better resource utilization, whether in high-tech centers or public institutions such as Brazil’s SUS (*Sistema Único de Saúde*).

#### 4.3.2. Methodological Constraints and Data Scope

The authors acknowledge several limitations. The apparent dip in 2025 reflects indexing delays, not a genuine decline in publication output. The analysis also focused on the 2014–2025 period and employed a controlled vocabulary of 15 standardized terms to ensure internal consistency and semantic precision. While this enhances methodological rigor, it limits representation of earlier historical literature.

This temporal focus was intentional. It captures the era in which next-generation methodologies—including FLASH radiotherapy, radiomics, circulating tumor DNA (ctDNA) profiling, and the Genomic-Adjusted Radiation Dose (GARD) framework—have matured. Collectively, these innovations define the contemporary phase of precision radiotherapy, where molecular insight and computational intelligence converge to guide clinical decision-making.

#### 4.3.3. Data Integrity and Model Sensitivity

The deliberate exclusion of non-DOI records and pre-2014 literature improved data quality and comparability but may have omitted historically important or recently under-indexed contributions. Additional limitations include potential language bias, as non-English articles were excluded, and uneven database coverage. Topic modeling also remains sensitive to parameter selection.

The absence of prospective validation using real-world clinical datasets is a key limitation for translational generalization. Even so, this methodological approach produced a clean, reusable baseline that can be expanded in future studies to cover longer temporal horizons.

Some thematic clusters—particularly Advanced Technologies—had limited sample sizes, resulting in wider uncertainty bands and validation challenges. Heterogeneous terminology and writing styles across sources further complicated semantic alignment, especially when processed by models such as Llama-3 (8B). These issues are intrinsic to large-scale, multilingual, and rapidly evolving biomedical corpora. They define the natural boundaries of first-generation integrative analyses.

### 4.4. Validation, Barriers, and Regulatory Frameworks

#### 4.4.1. Translational Gaps and Real-World Constraints

While the five-class system provides a theoretically complete framework for understanding precision radiotherapy, it still requires validation against direct clinical evidence. In this study, individual patients or clinical outcomes were not modeled. Instead, the analysis focused on organizing and synthesizing existing evidence at the conceptual and structural levels. Future work will link molecular and mechanistic variables—such as DNA repair capacity, tumor radiosensitivity, and biomarker expression—with clinical outcomes. These steps are essential for assessing the predictive utility, clinical reliability, and translational readiness of the framework.

#### 4.4.2. Ethical and Practical Barriers

From a translational perspective, several real-world barriers must be addressed before routine incorporation of this AI paradigm into clinical care. These include data heterogeneity across sites, limited interoperability between legacy systems (e.g., EHRs and PACS), and the lack of consistent regulatory frameworks for medical AI. Deep learning models often function as black boxes, which can undermine clinician trust. Explainable AI will be essential to provide transparency and ethical justification for model-driven decisions.

Standardization will also be critical for safe integration into radiotherapy workflows. Efficient computation must fit within clinical timeframes. Practical training for medical and technical staff will be necessary to ensure that innovation enhances, rather than compromises, safety and usability.

#### 4.4.3. Governance and Explainable AI

In parallel, regulatory agencies have strengthened requirements for AI deployment in healthcare, emphasizing transparency, accountability, and proven safety. For example, both the FDA and EMA now require that AI tools include detailed documentation of their purpose, underlying data, and validation methods. Interpretability and explainability have become mandatory rather than optional features. Governance frameworks such as the Comprehensive Algorithmic Oversight and Stewardship (CAOS) model and the Maturity Model for eXplainable Artificial Intelligence for Applied Engineering (MM4XAI-AE) are increasingly being adopted to guide documentation, validation, and monitoring procedures for responsible AI deployment [233,234,235,236,237,238,239,240,241,242].

Implementation of these requirements demands rigorous safeguards. Explainability tools—such as SHAP values, feature attributions, and heat maps—can provide clinicians with insight into how AI systems generate recommendations. Traceability mechanisms, including model cards, audit trails, and provenance tracking, enhance transparency across the entire model lifecycle. Finally, human-in-the-loop oversight ensures that clinicians remain integral to key decision processes, allowing automation to support rather than replace expert judgment [235,236,237,238,240,241,242,243,244,245].

Together, these practices can form the operational foundation for ethical governance and regulatory compliance. However, the path forward remains challenging. Deep learning models are inherently black-box systems and can never be fully interpretable. The diversity of imaging formats, clinical workflows, and local datasets further complicate external validation and limit real-world generalizability.

#### 4.4.4. Harmonization and Collaborative Oversight

The introduction of version control, bias monitoring, and post-deployment surveillance may place additional strain on clinical operations. Sustained collaboration between researchers, healthcare institutions, and regulatory bodies will be essential to develop harmonized validation standards and adaptive governance. With such collective effort, AI has the potential to transform precision radiotherapy into a practice that is safe, equitable, and sustainable [146,170,246,247,248,249,250,251,252,253].

### 4.5. Future Perspectives and Roadmap

#### 4.5.1. Methodological and Computational Advances

Looking ahead, the authors outline a clear roadmap for methodological advancement.

First, integrating physical and biological models of ultrafast radiotherapy with high-Z nanotechnologies could enhance system-level dynamic modeling.

Second, semantic supergraph analysis may identify underexplored research clusters, early emerging trends, and strategic opportunities for funding and collaboration.

Third, incorporating AI-derived biomarkers into adaptive, real-time radiotherapy planning will enable predictive systems to evolve alongside patient response, effectively closing the loop between inference and action.

#### 4.5.2. Clinical Translation and Adaptive Integration

Clinically, the proposed Precision Radiotherapy Implementation Plan (PRIP) should be viewed as a translational framework, not an immediately deployable protocol. At its current stage, it functions as a decision-support structure that organizes molecular, mechanistic, and radiobiological variables into adaptive planning pathways. Its clinical translation will depend on prospective validation across institutional datasets, alignment with hospital information systems (EHR, PACS, and TPS), and thorough regulatory and ethical review before routine application.

The Plan represents a foundational milestone bridging AI-driven semantic modeling and clinically actionable strategies. It serves as a mid-term translational objective designed to evolve into operational practice through validation, interoperability, and continuous learning within real-world settings.

#### 4.5.3. Toward a Learning Health Ecosystem

Integration of precision radiotherapy, biomarker-based personalization, and AI-based decision support marks a critical step toward measurable clinical impact. Recently published frameworks integrating molecular biomarkers, imaging analytics, and multi-omics data demonstrate that AI can translate these complex signals into actionable, individualized diagnoses and adaptive treatment plans. Collectively, these advances show that the convergence of biomarker-guided personalization and intelligent automation enhances accuracy, consistency, and clinical relevance in daily radiotherapy practice [169,170,254,255,256,257,258,259,260,261].

Taken together, this work represents a significant step forward in computational oncology. It demonstrates that combining diverse modalities in radiotherapy is not only feasible but potentially transformative when guided by clinical insight and public health priorities. By integrating heterogeneous data into a unified, decision-ready framework, this study moves the field closer to a continuously learning ecosystem—one in which semantic analytics, molecular understanding, and clinical innovation converge to deliver safer, more precise, and more equitable cancer care.

### 4.6. Historical and Analytical Context

These analyses describe how modern radiotherapy has evolved historically, semantically, and operationally into an interdisciplinary, data-driven ecosystem spanning radiobiology and oncology. The computational workflow began with automated retrieval from Scopus, PubMed, and Web of Science, followed by standardization, normalization, and metadata integration. This process yielded 3343 unique records.

The source imbalance, with PubMed as the primary contributor, reflects the field’s biomedical origin. The temporal profile of publication activity reveals three distinct phases: a low-output phase (1964–1990), a steady-growth phase (1991–2010), and a high-output phase (2011 onward). The most recent increase coincides with the development of stereotactic body radiotherapy, proton therapy, and predictive modeling. The apparent decline in 2025 most likely reflects indexing delay, not a real reduction in output.

A word cloud of titles and abstracts highlights recurrent terms—*cell*, *cancer*, *dose*, *treatment*, *radiotherapy*—underscoring the persistent interplay among mechanistic biology, dosimetry, and clinical practice. Latent Dirichlet Allocation (LDA) identified ten major topics across titles and abstracts, organized into two interrelated axes. The clinical–anatomical axis encompasses tumor sites, treatment modalities, and patient-centered outcomes. The mechanistic–molecular axis includes cellular responses, DNA repair, biomarkers, and toxicity pathways.

Together, these axes form a translational continuum where experimental findings and clinical needs inform one another iteratively. The co-occurrence supergraph displays high density (0.07), low modularity (0.15), and a clustering coefficient of 0.5, indicating strong semantic cohesion. The central node *cancer* connects directly to *tumor*, *radiotherapy*, *particle*, and *breast*, while technological subfields such as *proton*, *stereotactic*, and *imaging* occupy peripheral positions. These patterns suggest that methodological innovation anchors the system’s evolution at the boundaries of its conceptual space.

The findings demonstrate a deep conceptual convergence among biology, medical physics, and clinical care. Radiotherapy has transitioned from siloed subdisciplines to an integrated translational science. This model supports four working hypotheses.

First, *cancer* and *radiotherapy* form the structural axes of modern radiotherapy scholarship.

Second, the rising frequencies of *cell*, *dose*, *effect*, and *treatment* reflect a growing focus on personalization and clinical efficacy.

Third, the co-occurrence of *tumor*, *DNA*, *repair*, and *survival* indicates the increasing importance of precision medicine research.

Fourth, the observed high density and low modularity reveal the transversal and integrative character of the field.

#### 4.6.1. Thematic Domains and Case Analyses

Targeted database searches based on these hypotheses identified 61 highly relevant articles published between 2014 and 2025. These were grouped into five thematic domains:

(1) DNA repair and molecular responses;
(2) Precision oncology and genomic modeling;
(3) Individual radiosensitivity and clinical risk;
(4) Mechanisms of radioresistance;
(5) Advanced technologies and innovation in radiotherapy.

Case analyses illustrate how these themes complement each other. Studies on DNA repair focused on complex lesions from high-LET radiation, circadian synchronization of therapy, and modulation of the HR, NHEJ, ATM, and ATR pathways. Research on precision oncology integrated multi-omic data and AI-based models for dose calibration and genomic stratification, using indices such as RSI and GARD, and protein markers including RAD51, PARP1, CHK1, and MAPK15.

Studies on individual radiosensitivity combined genetic, molecular, and clinical data to generate personalized risk matrices using TCP/NTCP modeling, enriched with imaging, radiomics, and machine learning. Investigations of radioresistance highlighted the potential of high-LET modalities, repair inhibitors, and proteomic mapping to reverse resistant phenotypes. Research on advanced technologies reviewed the potential of FLASH radiotherapy and optimized brachytherapy to achieve tissue-sparing effects and **immune** modulation.

#### 4.6.2. Conceptual and Methodological Implications

Together, these studies established the foundation for the Integrated Implementation Plan in Precision Radiotherapy (IIPPR). This operational framework consists of five interdependent blocks that connect molecular discovery to translational validation and clinical delivery. It employs biomarker panels, genomic models, and adaptive protocols to support biologically informed treatment planning.

The plan was designed with Brazil’s Unified Health System (SUS) in mind. It defines measurable indicators—overall survival, progression-free survival, local control, grade ≥ 3 toxicity, unplanned hospitalizations, and average cost per controlled case—aligned with national health goals to enhance equity, reduce mortality, optimize resources, and expand access to advanced technologies.

Conceptually and methodologically, this study demonstrates that the integration of semantic mining, temporal modeling, and network analysis can reconstruct the evolution of a complex biomedical domain. Radiotherapy emerges as a coherent, interdependent system in which molecular biology, medical physics, and clinical decision-making reinforce one another within a continuous learning loop.

### 4.7. Limitations and Methodological Boundaries

The authors acknowledge several limitations. The apparent decline in 2025 reflects indexing lag. The analytic window (2014–2025) and exclusion of non-DOI records improved consistency and semantic precision but reduced historical depth. Heterogeneous terminology and limited published data for certain advanced technologies expanded the confidence intervals for some results. Nevertheless, Pearson correlations above 0.9 support the robustness of the main interpretations.

The implications of this work are significant. Theoretically, it provides a fine-grained conceptual map of modern radiotherapy. Operationally, it establishes a reproducible framework for implementing personalized, data-guided care pathways. For health policy, it outlines a scalable model for integrating advanced technologies into public healthcare systems.

Future directions include developing coupled physical and biological models of ultrafast radiotherapy integrated with high-Z nanoparticle platforms, expanding semantic supergraph analysis to uncover underexplored research clusters, and embedding AI-derived biomarkers into adaptive, real-time learning protocols. The convergence of semantic analytics, molecular modeling, and clinical innovation described here advances the vision of an ever-learning oncology ecosystem, capable of transforming data into safer, more precise, and more equitable cancer care.

## 5. Conclusions

This study demonstrates that AI-assisted semantic and temporal analysis of the scientific literature in radiotherapy, radiobiology, and oncology provides a robust and forward-looking foundation for a precision implementation agenda that bridges molecular science, clinical practice, and public health. By analyzing 3343 unique articles published between 1964 and 2025, the study reconstructs the historical trajectory and conceptual architecture of the field. It shows how radiotherapy has evolved from a fragmented research area into an integrated, interdisciplinary system characterized by strong semantic cohesion and translational continuity.

The results delineate a mature scientific landscape situated at the intersection of biology, physics, and clinical oncology. Strong semantic linkages were identified among the core concepts of *cancer*, *radiotherapy*, *DNA repair*, *radiation dose*, and *patient outcomes*. Two dominant research dimensions emerged. The clinical–anatomical axis is centered on tumor classification, therapeutic modalities, and prognosis. The mechanistic–molecular axis focuses on DNA repair pathways, biomarkers, and cellular responses to radiation.

The convergence of these dimensions underscores the translational character of the field. Molecular discoveries are increasingly being transformed into therapeutic and technological innovations with direct patient impact. Based on this integration, the study outlines the Integrated Implementation Plan in Precision Radiotherapy (IIPPR)—a structured model that translates scientific evidence into an iterative cycle of discovery, validation, and clinical deployment.

The program is organized into five operational classes:

1. DNA repair and molecular response.
2. Precision oncology and genomic modeling.
3. Individual radiosensitivity and clinical risk.
4. Mechanisms of radioresistance.
5. Innovative radiotherapy technologies.

Each class integrates biomarkers, predictive modeling, and therapeutic strategies to support the development of personalized treatment plans. The overarching objectives are to improve survival, reduce toxicity, and promote equitable access to advanced modalities such as FLASH-RT and hadron therapy.

The framework also addresses the long-standing problem of data silos in biomedical research. It harmonizes heterogeneous datasets through AI-based modeling, converting isolated findings into a coherent, decision-ready evidence base. In doing so, it positions AI as a central tool for interpreting and integrating the growing complexity of modern biomedical science.

Overall, the program suggests that radiotherapy, radiobiology, and oncology have achieved a high level of interdisciplinary maturity. This maturity supports the creation of a dynamic and integrated framework for precision medicine. Beyond computational synthesis, the program represents a conceptual advance in the organization and application of scientific knowledge. It transforms decades of accumulated data into a meaningful foundation for innovation, clinical decision-making, and the advancement of equitable cancer care.

## Figures and Tables

**Table 1:** Selected studies based on key expressions and thematic classification.

#	DOI	Title	Reference	Year	Terms Found	Classes *
1	10.1007/s12553-024-00895-y	Current trends and future perspectives in hadron therapy: radiobiology	[115]	2024	DNA repair	(1)
2	10.3389/fonc.2021.687672	The Time for Chronotherapy in Radiation Oncology	[116]	2021	DNA repair	(1)
3	10.1667/RADE-24-00037.1	What’s changed in 75 years of RadRes?—An Australian perspective on selected topics	[117]	2024	DNA repair	(1)
4	10.3389/fnano.2025.1603334	Radiobiological perspective on metrics for quantifying dose enhancement effects of High-Z nanoparticles	[118]	2025	DNA repair, precision oncology	(1, 2)
5	10.1016/j.clon.2021.09.003	Can Rational Combination of Ultra-high Dose Rate FLASH Radiotherapy with Immunotherapy Provide a Novel Approach to Cancer Treatment?	[119]	2021	flash radiotherapy	(5)
6	10.1016/j.canlet.2025.217895	Unraveling the dual nature of FLASH radiotherapy: From normal tissue sparing to tumor control	[15]	2025	flash radiotherapy, radiosensitivity	(5, 3)
7	10.18632/oncotarget.10996	Next generation multi-scale biophysical characterization of high precision cancer particle radiotherapy using clinical proton, helium-, carbon- and oxygen ion beams	[120]	2016	precision oncology	(2)
8	10.3390/ijms26136375	Radiation Therapy Personalization in Cancer Treatment: Strategies and Perspectives	[121]	2025	precision oncology	(2)
9	10.1080/14737140.2018.1458615	The effects of radiotherapy on the survival of patients with unresectable non-small cell lung cancer	[122]	2018	precision oncology	(2)
10	10.1016/j.hoc.2019.07.001	Toward a New Framework for Clinical Radiation Biology	[123]	2019	precision oncology	(2)
11	10.1016/S0007-4551(18)30383-7	Kidney cancer and radiotherapy: Radioresistance and beyond	[124]	2018	radioresistance	(4)
12	10.1093/jrr/rrz048	Carbon-ion irradiation overcomes HPV-integration/E2 gene-disruption induced radioresistance of cervical keratinocytes	[125]	2019	radioresistance, radiosensitivity	(4, 3)
13	10.1016/j.radonc.2022.08.001	Accurate prediction of long-term risk of biochemical failure after salvage radiotherapy including the impact of pelvic node irradiation	[126]	2022	radiosensitivity	(3)
14	10.1186/s13014-024-02566-8	Alpha/beta values in pediatric medulloblastoma: implications for tailored approaches in radiation oncology	[127]	2025	radiosensitivity	(3)
15	10.32471/exp-oncology.2312-8852.vol-43-no-3.16554	Can SARS-CoV-2 change individual radiation sensitivity of the patients recovered from COVID-19? (experimental and theoretical background).	[128]	2021	radiosensitivity	(3)
16	10.3390/cells12030360	Comparison of Radiation Response between 2D and 3D Cell Culture Models of Different Human Cancer Cell Lines	[129]	2023	radiosensitivity	(3)
17	10.2298/SARH220131085N	Current aspects of radiobiology in modern radiotherapy—our clinical experience	[130]	2022	radiosensitivity	(3)
18	10.1002/mp.13751	Genomics models in radiotherapy: From mechanistic to machine learning	[131]	2020	radiosensitivity	(3)
19	10.1016/j.rpor.2014.11.006	Hypersensitivity to chemoradiation in FANCA carrier with cervical carcinoma-A case report and review of the literature.	[132]	2015	radiosensitivity	(3)
20	10.3390/cancers8030028	Paradigm Shift in Radiation Biology/Radiation Oncology Exploitation of the H_2_O_2_ Effect for Radiotherapy Using Low-LET (Linear Energy Transfer) Radiation such as X-rays and High-Energy Electrons	[133]	2016	radiosensitivity	(3)
21	10.1259/bjr.20170949	Radiation biology and oncology in the genomic era	[134]	2018	radiosensitivity	(3)
22	10.1088/1361-6560/aac814	Radiobiological parameters in a tumor control probability model for prostate cancer LDR brachytherapy	[135]	2018	radiosensitivity	(3)
23	10.1136/bmjopen-2021-059345	The COMPLETE trial: HolistiC early respOnse assessMent for oroPharyngeaL cancEr paTiEnts; Protocol for an observational study	[136]	2022	radiosensitivity	(3)
24	10.1016/j.radonc.2023.109868	Voxel-based analysis: Roadmap for clinical translation	[137]	2023	radiosensitivity	(3)
25	10.1002/cnr2.1126	Progressive breast fibrosis caused by extreme radiosensitivity: Oncocytogenetic diagnosis and treatment by reconstructive flap surgery.	[138]	2019	radiosensitivity, DNA repair	(3, 1)
26	10.1002/mp.15177	Radiobiological comparison between Cobalt-60 and Iridium-192 high-dose-rate brachytherapy sources: Part I—cervical cancer	[139]	2021	radiosensitivity, hypoxia-induced	(3, 5)
27	10.1016/j.ijrobp.2015.11.013	Influence of Nucleoshuttling of the ATM Protein in the Healthy Tissues Response to Radiation Therapy: Toward a Molecular Classification of Human Radiosensitivity	[140]	2016	radiosensitivity, radioresistance	(3, 4)
28	10.3390/proteomes13020025	Deciphering Radiotherapy Resistance: A Proteomic Perspective	[141]	2025	radiosensitivity, radioresistance, DNA repair, precision oncology	(3, 4, 1, 2)

* Classes: (1) DNA repair, (2) Precision oncology, (3) Radiosensitivity, (4) Radioresistance, and (5) Flash radiotherapy.

**Table 2:** Major Biomarkers Associated with Tumor Progression or Regression after Therapy ^1^.

Category	Specific Biomarker	Mechanism/Clinical Interpretation	Detection Modality	Associated Cancers	Predictive Performance	Limitations	References
**Circulating Tumor DNA (ctDNA)**	ctDNA (absolute levels or tumor fraction)	Early reduction → regression; increase/persistence → progression or resistance	Targeted sequencing/plasma NGS	NSCLC, colorectal, breast, gastroesophageal	High sensitivity for early detection of response or resistance (days to weeks before imaging)	Low sensitivity in “low-shedding” tumors; dependence on personalized panels	[206]
	ctDNA—Variant Allele Frequency (VAF)	Rapid VAF decline after therapy onset → favorable response	ddPCR or NGS	NSCLC, melanoma, colorectal	Strong correlation with PFS and OS	Affected by tumor heterogeneity and clonal evolution	[207]
	ctDNA—emerging resistance mutations (e.g., EGFR C797S)	Detection of resistance mutations → impending therapeutic failure	Longitudinal sequencing	NSCLC	High specificity for acquired resistance	Requires prior knowledge of mutational profile	[208]
	ctDNA—personalized “fingerprint” panels	Multi-mutation tracking per patient → enhanced coverage of tumor heterogeneity	WES + customized panels	Multiple solid tumors	Better sensitivity vs. fixed panels	Higher cost and logistical complexity	[209]
**Epigenomic/Extracellular Vesicle Markers**	cfDNA methylation profiles	Distinct methylation patterns linked to molecular subtypes and immune response	Bisulfite sequencing or methylation arrays	NSCLC, colorectal	High prognostic accuracy; predicts immunotherapy response	Requires advanced bioinformatics	[210]
	EV-miRNAs (extracellular vesicle miRNAs)	Post-transcriptional regulation of immune and oncogenic pathways	EV RNA-seq or qPCR	NSCLC, breast	Complements cfDNA; enhances stratification	Low stability; isolation variability	[210]
**Proteomic/Serum Markers**	sHER2, CEA, CA15.3	Serum levels correlate with tumor burden and treatment response	ELISA/immunoassay	Breast, colorectal	Useful for continuous monitoring; low cost	Limited specificity when used alone	[211]
	Plasma proteomic signatures (aptamer-based)	Dynamic profiles reflect tumor activity and immune response	Aptamer proteomics (e.g., SomaScan)	NSCLC	High temporal resolution; predicts immune-related adverse events (irAEs)	Requires broad clinical validation	[212]
	Cytokeratins, TK1, growth factors	Indicators of proliferation and aggressiveness	Immunoassay	Colorectal	Associated with stage and prognosis	Less sensitive than ctDNA	[213]
**Immunologic Markers**	Tumor-infiltrating lymphocytes (TILs)	High density → regression; exhaustion → progression	Tissue IHC or RNA-seq	Pan-cancer	Robust prognostic value	Invasive; not suitable for serial monitoring	[214]
	T-cell receptor (TCR) repertoire	Early clonal expansion → immunotherapy response	Blood TCR sequencing	Melanoma, HNSCC	Predictive for immune checkpoint blockade	Analytical complexity	[215]
	Ferroptosis-related gene activity	Ferroptotic death → enhanced immunogenicity	Transcriptomic scoring (ferroptosis score)	NSCLC, melanoma	Adds value beyond TMB/PD-L1	Mechanism under functional validation	[216]
	*PTPRD/PTPRT* mutations	Loss of function → inflammatory tumor microenvironment → better ICB response	Genomic sequencing	Pan-cancer	Genomic biomarker for pan-cancer response	Variable frequency among tumors	[217]
**Imaging Biomarkers**	Early Tumor Shrinkage (ETS)	≥20% reduction within 6–8 weeks → regression	CT/MRI (RECIST)	Colorectal, NSCLC	Correlates with PFS/OS	Limited by pseudoprogression or stable disease	[218]
	Depth of Response (DpR)	Maximum tumor shrinkage → improved prognosis	Continuous imaging analysis	Various solid tumors	More informative than binary response	Depends on standardized imaging protocols	[218]
**Genomic/Transcriptomic Signatures**	Chromosomal instability (CIN)	High CIN → poor prognosis	Gene expression profiling (e.g., Carter et al.)	Breast, ovarian, colorectal	Independent prognostic value	Static; based on single biopsy	[219]
	Progression Gene Signatures (PGS)	Activation of proliferation/metastasis pathways → progression	RNA-seq + ML modeling	Multiple cancers	High predictive power in cohorts	Requires prospective validation	[220]
	Kinome profiling	Activation of tumor-promoting kinase pathways	Functional proteomics + RNA	Colon	Identifies therapeutic targets	Technically complex; limited clinical use	[221]
**Integrated Models**	ctDNA + imaging	Combines molecular dynamics with anatomical response	ML-based multimodal integration	Breast, NSCLC	Superior predictive accuracy vs. single biomarkers	Requires computational infrastructure	[222]
	ctDNA + proteomics + immunophenotyping	Comprehensive profile of tumor–host evolution	Longitudinal multi-omics	NSCLC	High predictive and stratification accuracy	High cost; lack of standardization	[210]

^1^ Circulating tumor DNA (ctDNA) remains the most sensitive early indicator of patient response to therapy. Decreases in absolute ctDNA levels or tumor fraction indicate tumor regression. Stable or rising levels suggest tumor progression or the emergence of resistance. Changes in variant allele frequency (VAF) correlate strongly with progression-free and overall survival in non-small cell lung cancer (NSCLC), colorectal cancer, and melanoma. However, sensitivity is reduced by tumor heterogeneity. As a result, patient-specific fingerprint panels derived from whole-exome sequencing are often required. The appearance of known resistance mutations, such as EGFR C797S, provides a clear indication of therapy failure. Epigenomic ctDNA methylation profiles and extracellular vesicle (EV)-derived miRNAs show strong prognostic and predictive value for immunotherapy in NSCLC and colorectal cancer. These analyses, however, depend on advanced machine learning and complex bioinformatics pipelines and can be affected by inter-laboratory variability. In contrast, proteomic and serum markers such as sHER2, CEA, and CA15.3 are inexpensive and widely available, but their individual specificity is limited. Recently developed plasma proteomic signatures, including those measured by SomaScan, offer higher temporal resolution and may predict immune-related adverse events. Although promising, these signatures still require validation in large prospective cohorts. Tumor-infiltrating lymphocytes (TILs) and T-cell receptor (TCR) repertoire features consistently predict response to immunotherapy across multiple cancer types. However, serial monitoring of these parameters over time remains technically challenging. Emerging biomarkers such as ferroptosis-related gene activity and PTPRD/PTPRT mutations provide additional mechanistic insight into tumor immunogenicity and checkpoint blockade response, potentially refining patient selection. Imaging biomarkers, including early tumor shrinkage (ETS) and depth of response, are strongly associated with survival in NSCLC and colorectal cancer. These measures can, however, be confounded by pseudoprogression or prolonged stable disease in immunotherapy settings. Similarly, genomic and transcriptomic signatures, such as chromosomal instability, progression gene sets, and kinome profiles, offer valuable prognostic and mechanistic insights but are often static and limited by single-biopsy sampling. The most significant advances have emerged from integrative multi-omics approaches that combine ctDNA kinetics with proteomic, immunophenotypic, and imaging features using machine learning. These frameworks enable real-time adaptive decision-making and have shown early success in NSCLC and breast cancer. They improve prediction and patient stratification but remain constrained by cost, standardization, and the need for rigorous clinical validation. Overall, dynamic and minimally invasive biomarkers—particularly ctDNA and serum proteomics—are becoming the cornerstone of precision oncology. Their greatest impact will likely occur within multi-modal frameworks that integrate molecular, immune, and imaging data to address tumor heterogeneity and the continuously evolving immune microenvironment.

**Table 3:** Integrated Precision Radiotherapy Plan.

Indicator	Related Plan Class(es)	Description and Measurement Method	Expected Public Health Impact
Overall survival (OS) at 1, 3, and 5 years	2, 3, 4, 5	Percentage of patients alive 1, 3, and 5 years after radiotherapy, stratified by tumor type and treatment protocol.	Improved longevity and reduction in cancer-specific mortality, particularly in high-risk tumors.
Progression-free survival (PFS)	2, 3, 4, 5	Mean time to recurrence or disease progression after personalized radiotherapy.	Greater disease control, reduced retreatment, and lower hospital costs.
Local and regional control ratel	1, 2, 3, 4	Proportion of patients without recurrence in the tumor bed or regional lymph nodes after 12 and 24 months.	Increased therapeutic efficacy and improved population prognosis.
Incidence of acute and late toxicities (grade ≥ 3)	1, 2, 3	Standardized reporting (RTOG/EORTC or CTCAE) of severe adverse events related to radiotherapy.	Reduced disabling side effects and improved post-treatment quality of life.
Rate of unplanned hospitalizations related to treatment	1, 3	Percentage of patients requiring emergency hospitalization due to radiotherapy complications.	Savings in hospital resources and reduced burden on inpatient units.
Percentage of patients undergoing molecular and functional stratification prior to treatment	1, 2, 3	Proportion of patients who underwent genomic, proteomic, or functional testing before radiotherapy initiation.	Greater precision in therapeutic planning and optimization of resource allocation.
Proportion of treatments with adaptive protocols	2, 3, 5	Percentage of patients whose radiotherapy plan was adjusted during treatment based on clinical response and/or biomarkers.	Reduced toxicity, improved efficacy, and more rational use of technology.
Complete and partial response rate	2, 4, 5	Percentage of patients with complete disappearance or significant reduction in tumor after treatment.	Direct indicator of personalized protocol effectiveness.
Average cost per patient with tumor control achieved	2, 5	Total treatment cost divided by the number of patients with sustained complete or partial response.	Increased economic efficiency of the public health system.
Mean hospital stay due to treatment complications	1, 3	Average number of days of hospitalization caused by radiotherapy-related adverse events.	Reduced hospital bed usage and expanded treatment capacity.
Geographic coverage of services with multi-omic and radiobiological capacity	1, 2, 3	Percentage of health regions with infrastructure for multi-omic testing and radiobiological integration.	Reduction in regional inequalities in access to advanced treatments.
Percentage of patients with access to advanced therapies in the public health system (SUS)	4, 5	Proportion of patients receiving modalities such as FLASH-RT, optimized brachytherapy, or particle therapy.	Equity in access to innovative technologies across the national territory.
Proportion of cases integrated into national registries with molecular and radiomic data	1, 2, 3, 4, 5	Percentage of patients with clinical, molecular, and imaging data stored in national repositories.	Strengthened epidemiological surveillance and translational research.
Utilization rate of machine learning in clinical planning	2, 3, 5	Percentage of cases planned or adjusted using validated AI models.	Standardization of evidence-based practices and increased outcome predictability.
Number of adaptive protocols validated and incorporated into national guidelines	3, 4, 5	Number of adaptive clinical protocols formally approved and implemented in the public health system.	Continuous improvement of care quality and systematic technological updating.

## Data Availability

The data supporting the findings of this study are available from the corresponding author upon reasonable request.

## Abbreviations

The following abbreviations are used in this manuscript:

Abbreviation/Term	Meaning	Context/Explanation
≥3 Toxicity	Grade 3 or Higher Adverse Events	Represents severe clinical side effects per standardized toxicity grading.
≥40 Gy/s	Dose-Rate Threshold for FLASH-RT	Represents ultrafast radiation rate preserving normal tissue integrity.
0.291–0.669 (~0.378 span)	Range of Observed Cosine Scores	Captures the minimum-to-maximum similarity observed between LLM outputs.
0.5235 ± 0.0271 (95% CI 0.4964–0.5506)	Aggregate Similarity Statistics	Demonstrates moderate overall alignment and high reproducibility of language/semantic structure between models.
15 Standardized Terms	Controlled Vocabulary Set	Used for harmonizing semantic meaning across Scopus, PubMed, and Web of Science records.
2014/2015–2024 windows	Temporal limits of corpus	Ensure focus on contemporary, translational radiotherapy.
2014–2025 Analytic Window	Defined Temporal Scope of Corpus	Restriction ensuring semantic stability and consistent term usage across databases.
2D/3D	Two-dimensional/Three-dimensional	Cell-culture models compared for radiation response.
3D Spheroids	Three-Dimensional Cell Cultures	In vitro tumor analogs reproducing microenvironmental gradients and resistance.
60Co/^192^Ir	Cobalt-60/Iridium-192 Isotopes	Common brachytherapy sources; compared for efficacy, RBE, and safety.
a	Abstracts Corpus	The suffix “a” in *Topic_0a*–*Topic_9a* refers to topic models generated from abstracts rather than titles.
Adaptive Clinical Protocols	Dynamically Updated Treatment Guidelines	Clinical pathways that adjust based on biomarker or imaging feedback.
Adaptive Radiotherapy Planning	Treatment Optimization Based on Patient Response	Future goal using AI-derived biomarkers for closed-loop, real-time dose adjustment.
Adjacency definition	Mathematical rule for linking nodes	Directed adjacency alters numeric scales of network metrics.
Advanced Radiotherapy Technologies	Emerging Modalities (FLASH-RT, Brachytherapy, Particle Therapy)	High-precision and/or ultrafast radiation technologies providing enhanced therapeutic index.
Advanced Technologies Cluster	Thematic Category in LDA Model	Identified area with limited samples causing wider uncertainty in validation.
AGENT	Therapeutic Agent	Denotes a drug, molecule, or compound used in combination with radiation therapy.
AI	Artificial Intelligence	Central methodological pillar of the study; refers to machine-learning and computational modeling approaches.
AI Models (Validated)	Certified Predictive Algorithms	Used to plan or adjust treatments, ensuring regulatory and scientific reliability.
AI synthesis	Artificial Intelligence synthesis	Refers to AI-based interpretation and summarization phase of the pipeline.
AI-assisted	Artificial Intelligence–Assisted	Denotes the use of AI in processing, interpreting, and modeling scientific literature.
AI-based	Artificial Intelligence-based	Refers to the study’s computational modeling methods.
AI-based methods	Artificial-intelligence analytical techniques	Include topic modeling, text mining, and co-occurrence network analysis.
AI-based Modeling	Artificial Intelligence–Based Modeling	Framework for harmonizing heterogeneous datasets into unified evidence models.
AI-derived Biomarkers	Machine-Learned Predictive Molecular Indicators; Artificial Intelligence–Generated Predictive Features	Proposed for real-time, adaptive radiotherapy planning linked to patient-specific biological response; used for adaptive treatment and real-time learning protocols.
Alpha/Beta (α/β)	LQ model tissue parameters	Reported in pediatric medulloblastoma for tailoring dose/fractionation.
ATM	Ataxia-Telangiectasia Mutated	DNA-damage response kinase; nucleoshuttling linked to tissue radiosensitivity.
ATR	Ataxia Telangiectasia and Rad3-Related Protein	Parallel kinase to ATM; monitors replication stress and coordinates DNA repair.
Average Degree = 9.5	Mean number of links per node	Quantifies average lexical connectivity.
Average Weighted Degree = 1962.2	Mean sum of edge weights	Captures total co-occurrence frequency per term.
BER	Base Excision Repair	DNA repair pathway correcting small, non-helix-distorting base lesions caused by ionizing radiation.
BIOLOGICAL	Biological Effect or Endpoint	Used in *Topic_2a* for biophysical modeling linking energy deposition to cellular effect.
Biomarker signature	Composite molecular indicator	Selection criterion for case-study inclusion and stratification.
Biomedical Stopwords	Domain-specific uninformative terms	Filtered to improve semantic precision.
Biomedical subject areas	Domain filter in Scopus	Restricts search to clinically relevant literature.
Blue–Red Color Bar	Visual encoding of co-occurrence strength	Blue = weak association; red = strong association.
BMAL1, CLOCK, PER, CRY	Core Circadian Genes	Regulate cellular timing of DNA repair; targeted in chronoradiotherapy to reduce toxicity.
Brachytherapy	Internal radiotherapy using implanted sources	Another advanced modality considered in the framework.
Brachytherapy dose escalation	Targeted increase in internal RT dose	Inclusion term; linked to voxel-level and TCP modeling.
Carcinoma	Cancer Type Originating from Epithelial Cells	Frequently used term in radiation oncology literature (breast, esophageal, prostate).
CD8^+^ Lymphocytes	Cytotoxic T Cells	Immune effectors preserved after FLASH-RT, supporting anti-tumor immunity.
Cell-Fit-HD	High-Density Cell Microbeam Technology	Experimental system quantifying DNA-damage kinetics and repair precision.
Central node	Highest-connectivity vertex	“Cancer” identified as the core of the radiotherapy supergraph.
cGAS–STING	Cyclic GMP–AMP Synthase/Stimulator of Interferon Genes	Cytosolic DNA-sensing pathway linking radiation damage to immune activation.
ChatGPT	Generative Pretrained Transformer (GPT-5 model, OpenAI)	AI-based language model used under human supervision to improve clarity, grammar, and precision during manuscript preparation.
Chronoradiotherapy	Time-Synchronized Radiation Delivery	Aligns irradiation with circadian repair peaks to enhance efficacy and minimize side effects.
CI	Confidence Interval	Statistical interval representing uncertainty around the mean cosine-similarity estimates (95% CI: 0.4964–0.5506).
CiteSpace	Citation Space Mapping Tool	Bibliometric software for co-authorship and trend analysis.
Class 1—DNA Repair and Molecular Response	Class 1 Category	Focused on DNA damage signaling, repair pathways (HR, NHEJ, NER, BER), and checkpoint activation; aligns molecular biology with therapeutic personalization.
Class 2—Precision Oncology and Genomic Modeling	Class 2 Category	Integrates genomic profiling, machine-learning models, and patient stratification to personalize radiation therapy.
Class 3—Individual Radiosensitivity and Clinical Risk	Class 3 Category	Addresses variability in patient radiation response, genetic predisposition, and predictive biomarkers.
Class 4—Radioresistance and Associated Mechanisms	Class 4 Category	Explores resistance mechanisms (hypoxia, metabolism, stemness, viral integration) and corresponding therapeutic interventions.
Class 5—Advanced Technologies and Innovative Radiotherapy	Class 5 Category	Encompasses next-generation technologies including FLASH-RT, hadron therapy, voxel-based analysis, and high-Z nanoparticle radiosensitization.
CLINICAL/ONCOLOGY/RADIOBIOLOGY/MEDICAL/DEVELOPMENT/ELSEVIER/RIGHT/RESERVED	Publishing or Institutional Terms	*Topic_8a* includes frequent non-scientific tokens derived from editorial metadata, likely due to text-parsing of abstracts from publishers (Elsevier, etc.).
Clinical trial phase	Trial stage indicator	Used to filter translational maturity of studies.
Clinical–Anatomical Axis	Axis describing patient- and site-specific features; dimension grouping disease sites, therapies, and outcomes	Encompasses disease sites, therapy types, outcomes; derived from topic modeling to describe clinical themes.
Clustering coefficient	Likelihood that connected nodes form closed triangles	High value shows local semantic cohesion.
Co-Occurrence Supergraph	Graph of term co-appearances; combined network of all significant co-occurring terms	Reveals structural relationships among concepts in the literature; integrates molecular, clinical, and technological vocabularies into one semantic structure.
Combined modality	Multimodal treatment (e.g., RT + IO)	Inclusion term bridging lab and clinic.
COMPLETE (trial)	HolistiC early respOnse assessMent for oroPharyngeaL cancEr paTiEnts	Protocol integrating imaging and early-response assessment in oropharyngeal cancer.
COMPLETE Protocol	Combined Multiparametric Imaging, Radiomics and Machine-Learning Framework	Integrates imaging and omics data for personalized radiotherapy planning.
Computational Oncology	Field Integrating AI and Cancer Research	The disciplinary frame under which the study’s integrative modeling approach is positioned.
COPPE	Instituto Alberto Luiz Coimbra de Pós-Graduação e Pesquisa de Engenharia	UFRJ’s graduate engineering institute; associated with the Nanotechnology Engineering Program.
Cosine Similarity	Vector-Space Similarity Measure	Quantifies the semantic and lexical overlap between two text representations (range 0–1). Used here to assess alignment between outputs of different LLMs.
Cost per Controlled Case	Economic Indicator	Total treatment cost divided by number of patients with complete or partial sustained response; measures cost-effectiveness.
COVID-19	Coronavirus Disease 2019	Systemic factor influencing radiosensitivity and patient management.
Cross-Model Consistency	Comparative Validation Concept	Indicates methodological reliability by reproducing semantically equivalent results across distinct LLM architectures.
CSV	Comma-Separated Values	Log format for timestamped model inferences and auditing.
CTCAE	Common Terminology Criteria for Adverse Events	Global clinical-trial standard for grading radiotherapy-related adverse events.
Data Repositories	Centralized Databases for Clinical and Molecular Information	National archives enabling interoperability and machine-learning-based outcome prediction.
DAY	Treatment Day/Fraction Day	Represents time variable in radiotherapy fractionation (*Topic_7a*).
Decision-Ready Framework	Actionable Computational Infrastructure	Describes the output: a structured knowledge base usable for policy, funding, and clinical translation.
Deduplication	Removal of duplicate entries	594 records removed by ID and 25 by title → 3343 unique publications.
Density	Ratio of existing to possible edges; ratio of actual to possible edges	0.5 in the directed setup, signifying a tightly connected lexical field; Indicates a dense and cohesive co-occurrence network.
DER/SER/RBE	Dose Enhancement Ratio/Sensitization Enhancement Ratio/Relative Biological Effectiveness	Quantitative radiobiological indices comparing biological responses between radiation modalities.
Diameter	Longest shortest path in the network; Longest shortest-path length	Here equals 1 due to directed adjacency; reflects condensed connectivity; reflects immediate connectivity among principal terms in a directed adjacency setup.
Directed/Weighted Graph	Graph with edge orientation and numerical weights	Models term co-occurrence frequency and directionality in the semantic network.
DNA	Deoxyribonucleic Acid	Central mechanistic concept related to damage, repair, and radiosensitivity.
DNA Repair	Deoxyribonucleic Acid Repair	Core biological process for maintaining genomic integrity after radiation exposure; major research theme representing molecular response to radiation.
DOI	Digital Object Identifier	Persistent identifier ensuring record uniqueness and traceability.
Dose–Response Model	Quantitative relation between dose and effect; mathematical link between dose and biological effect	Appears among inclusion keywords; supports mechanistic theme; underpins LDA topics and clinical correlation analysis.
E2	HPV Regulatory Protein	Loss disrupts viral genome control; restoration of E2-associated checkpoints observed with carbon-ion irradiation.
Edge Thickness	Graph-visualization parameter	Increases proportionally with co-occurrence weight.
Editorial Workflow (“Llama → GPT-4o”)	Sequential Model Use	Denotes the pipeline where Llama3 generated drafts and GPT-4o refined phrasing, without altering data or analytic interpretations.
Effect	Biological or Clinical Outcome	Used to represent both therapeutic effects and adverse effects of radiation exposure.
ENERGY	Beam Energy	Physical parameter determining penetration depth and RBE of particle beams (*Topic_2a*).
EORTC	European Organisation for Research and Treatment of Cancer	European body with harmonized toxicity and clinical-trial reporting frameworks.
Eq.	Equation	Used to identify model equations from Equations (A11)–(A20).
Equations (A1)–(A20) (Appendix A and Appendix B)	Numerical LDA Output Tables	Provide word probabilities and model parameters for transparency.
Equity/Access Metrics	Health Policy Indicators	Includes survival, toxicity, cost per controlled case, and hospitalizations for public health alignment.
EXPRESSION/GENE/PROTEIN/BLOOD	Biomarker-related Terms	In *Topic_6a*, these describe molecular and clinical biomarkers used for radiosensitivity prediction.
Factor	Biological or Statistical Factor	Represents either biological modifiers (e.g., hypoxia) or regression factors in predictive models.
FANCA	Fanconi Anemia Complementation Group A	Germline carrier state linked to hypersensitivity to chemoradiation.
Feasibility/Translational Potential	Applied Assessment Metrics	Used to evaluate how physical models and clinical data can be realistically implemented within current technological and clinical constraints.
Feature Space	Multidimensional Representation of Texts	Conceptual domain in which cosine similarity evaluates orientation and semantic overlap between document vectors.
FIU	Florida International University	U.S. collaborator institution for computational and AI analysis.
FLASH	Ultrafast Radiotherapy (FLASH-RT); Ultrafast radiotherapy at ultra-high dose rates; ultrafast (>40 Gy s^−1^) radiation-delivery technique; Ultrafast (≥40 Gy s^−1^) radiotherapy	High-dose-rate radiation mode producing reduced normal-tissue toxicity; investigated for normal-tissue sparing and tumor control; one of the advanced technologies highlighted in Class 5; modern modality delivering high-dose-rate pulses with reduced normal-tissue damage.
FLASH Radiotherapy and Brachytherapy	Advanced Techniques	Explored for synergistic effects in tissue protection and immune modulation.
FRACTION	Dose Fraction	In *Topic_9a*, refers to division of total dose into multiple treatment sessions—key variable in radiobiological modeling.
Fraction/Fractionation	Division of Total Radiation Dose into Multiple Sessions	Appears among frequent title/abstract terms; optimization variable flagged by abstracts analysis.
Frequency Trimming	Vocabulary Cutoff Procedure	Eliminates extremely rare or overly common tokens.
G ≥ 3	Grade 3 or Higher Toxicity	Severe adverse-event category per CTCAE or RTOG/EORTC scales; measures safety profile.
GARD	Genomic Adjusted Radiation Dose	Integrates RSI and gene-expression data to personalize dose prescription.
Genomic/Proteomic Modeling	Molecular Data Integration Approaches	Computational modeling of genomic (gene-level) and proteomic (protein-level) datasets to predict radiosensitivity and outcomes.
Genomic Classifier	Model Assigning Molecular Subtypes	Used for precision oncology decisions.
Genomic Modeling	Predictive Modeling Based on Genetic Data; Mechanistic to Machine-Learning Frameworks	Used for individualized therapy planning in precision oncology; used to personalize radiotherapy (predict response/toxicity).
Gephi	Open-source Network-Analysis Software	Used for calculating density, diameter, clustering, and modularity of the co-occurrence network.
GPT-4o	OpenAI’s Multimodal Large Language Model (Omni Version)	Used to refine language, maintain consistency, and ensure terminological precision; served only in editorial and validation steps.
GPT-5	Generative Pretrained Transformer, Version 5	Model used within ChatGPT for generative text refinement and consistency checking.
Grammarly (v.2025)	AI-Powered English Language Writing Assistant	Utilized for grammatical and stylistic correction to ensure readability for international readers.
Gy	Gray	SI unit of absorbed radiation dose (1 Gy = 1 J/kg); frequent in topics 4a, 7a, 9a related to dosimetry and toxicity.
Gy/Gy s^−1^	Gray/Gray per second	SI units of absorbed dose and dose rate.
H_2_O_2_	Hydrogen Peroxide	Central to radiosensitization concepts exploiting oxidative effects (low-LET contexts).
Hadron Therapy	Particle-Beam Radiotherapy (Protons, Carbon Ions); Particle-Based Radiotherapy (Using Protons or Heavy Ions)	Emerging high-precision radiation technique; advanced technology referenced in the framework as part of “Innovative Radiotherapy Technologies.”
Head and Neck/Breast/Prostate Cancer	Disease-Site Descriptors	Define clinical–anatomical axes in *Topic_0t* and related clusters.
Heavy Charged Particles	Proton/Helium/Carbon/Oxygen Ion Beams	“Next-generation multi-scale” characterization and high-LET effects.
HIF	Hypoxia-Inducible Factor	Transcription factor activated by VHL loss; mediates hypoxia-related radioresistance.
High-End Modalities	Advanced Treatment Technologies	Includes FLASH-RT, hadron therapy, and other precision radiotherapy innovations.
High-LET	High Linear Energy Transfer	Radiation that deposits dense energy tracks (e.g., heavy ions); generates complex DNA lesions.
High-LET Modalities	Heavy-Ion or Particle Therapies	Used in Class 4 to reverse resistant phenotypes via dense-ionization damage.
High-LET Radiation	High Linear Energy Transfer Radiation	Mentioned indirectly in prior results, relevant here as part of the “system-level dynamic modeling” refinement.
High-Z	High Atomic-Number Elements	Nanoparticles used to enhance dose deposition and radiosensitization.
High-Z Nanoparticles	High Atomic-Number Nanomaterials	Act as radiosensitizers by amplifying local dose and reactive-species generation.
High-Z Nanotechnologies	High Atomic Number Nanomaterials	Proposed future pathway to couple with ultrafast radiotherapy for dose amplification and radiosensitization.
High-Z Platforms	High Atomic Number Platforms	Refers to nanomaterials designed to enhance radiotherapy through dose amplification.
Hospitalization Rate	Frequency of Radiotherapy-Related Admissions	Indicator of adverse-event management effectiveness.
HPV	Human Papillomavirus	Oncogenic virus associated with cervical-cancer radioresistance; carbon-ion therapy shown to overcome it.
HPV/E2	Human Papillomavirus/Regulatory Gene	Integration/E2 disruption associated with cervical radioresistance; carbon ions reported to overcome it.
HR	Homologous Recombination	Accurate double-strand-break repair pathway; its inhibition increases radiosensitization.
Hybrid Modeling/Model Fusion	Combined Output Strategy	Not used in this study; models were compared for agreement, not merged to produce shared outputs.
Hypotheses 1–4	Structural Inferences Derived from Topology	Define conceptual axes: (1) cancer + radiotherapy centrality; (2) personalization trends; (3) biomarker emphasis; (4) transversal field integration.
ID Numbers	Unique Database Identifiers (e.g., PMID, DOI, EID)	Used for automated deduplication across sources.
IIPPR	Integrated Implementation Plan in Precision Radiotherapy	The overarching operational and translational framework structuring radiotherapy research into five classes with progressive integration into health policy.
IMA	Instituto de Macromoléculas Professora Eloisa Mano	UFRJ institute specializing in polymers and macromolecular science.
Imaging	Medical Imaging	Used for diagnosis, planning, and verification in radiotherapy (e.g., MRI, CT, PET).
Immunoradiotherapy	Radiotherapy Combined with Immune Modulation	Emergent interdisciplinary theme.
Immunotherapy	Immune-Based Cancer Therapy	Appears in *Topic_5a*; integration of radiation with immunotherapy strategies.
In vitro	Laboratory-Based Experiments	Denotes cellular studies that feed mechanistic insight.
Individualized Radiosensitivity Assessment	Patient-Specific Radiation Response Evaluation	Quantifies how individual genetic and biological profiles modify treatment tolerance and efficacy.
Integrated Implementation Plan in Precision Radiotherapy (IIPPR)	Translational Framework	Unifies molecular, clinical, and technological insights into five interdependent operational blocks for precision oncology and public-health integration.
Interdisciplinary Maturity	Cross-Disciplinary Integration	Refers to the field’s evolution into a coherent system combining biology, physics, and clinical practice.
Ion	Ion Beam	Refers to charged particle beams (proton, helium, carbon, oxygen) used in particle radiotherapy.
ION/PARTICLE/PROTON/CARBON/PHOTON	Ion-Beam and Particle Radiation Terms	Terms appearing together in *Topic_2a*; describe physical particles used in hadron therapy.
Irradiation	Exposure to Ionizing Radiation	Common in Topics 6a and 7a; refers to experimental or clinical exposure processes.
k = 10 Topics	Number of LDA Topics	Defines model granularity; ten topics selected for interpretability and stability.
Kidney Cancer VHL/HIF Axis	von Hippel–Lindau/Hypoxia-Inducible Factor	Mechanistic route to pseudohypoxia and resistance.
KORTUC II	Kochi Oxidative Radiotherapy for Unresectable Carcinomas Type II	Technique using hydrogen peroxide (H_2_O_2_) to increase tumor oxygenation and radiosensitivity.
LaBioS	Laboratório de Biopolímeros e Sustentabilidade	Laboratory supporting the experimental and organizational phases of the study.
LabOPTIMA	Laboratory for Optimization, Data Analytics, and Artificial Intelligence in Materials Science	UFRJ-based lab responsible for data-centric analysis and computational modeling; mentioned as a contributor to data processing and validation.
LDA	Latent Dirichlet Allocation	Unsupervised topic-modeling algorithm used for semantic extraction of dominant research axes.
LDR	Low-Dose-Rate	Brachytherapy context (e.g., prostate cancer TCP modeling).
LDR Brachytherapy Sources ^60^Co/^192^Ir	Cobalt-60/Iridium-192	Compared radiobiologically (e.g., cervical cancer HDR; RBE differences).
Lemmatization	Word-Form Normalization	Reduces inflectional variants to base forms for stable modeling.
LET	Linear Energy Transfer	Energy deposition per track length; key in radiobiology and particle therapy.
LEVEL	Expression Level/Dosimetric Level	Indicates quantitative measurement of gene, protein, or dose levels (*Topic_6a*).
LINE	Cell Line	Experimental model system used for in vitro radiation studies (*Topic_1a*).
Linear	Linear Model (Linear-Quadratic Model)	Refers to the linear component of the LQ model used to describe cell survival curves in radiotherapy modeling.
Llama-3 (8B)	Large Language Model (8 Billion Parameters); Open-Source Large Language Model (8 Billion Parameters)	Referenced as a semantic-processing model used during analysis, highlighting technical limitations and multilingual challenges; used for initial draft generation and offline semantic analysis; suitable for secure, local computation.
LLM	Large Language Model	AI model trained on massive text corpora; in this study, Llama-3 (8B) and GPT-4o were compared.
LLMs	Large Language Models	Used for semantic synthesis under fixed prompts; outputs logged for auditability; models (like GPT-type) used in PaperProcessor for semantic synthesis and summarization.
Local Control/Systemic Control	On-Site vs. Distant Tumor Suppression	Metrics evaluating therapeutic success at tumor site and metastasis prevention.
Machine Learning	AI Technique for Pattern Recognition and Prediction	Supports risk stratification, biomarker identification, and outcome modeling.
Mean (μ)	Arithmetic Average	Represents central tendency of similarity values (~0.52 overall).
Mean Clustering Coefficient = 0.5	Local Cohesion Metric	Reveals that many terms form tightly knit clusters (≈1140 triangles).
Mechanism/Molecular	Mechanistic and Molecular Basis	Indicates interest in the cellular and sub-cellular mechanisms of radiation injury and repair.
Mechanistic–Molecular Axis	Axis Describing Biological Mechanisms; Dimension Encompassing DNA Repair and Biomarkers	Encompasses cellular responses, DNA repair, and biomarkers; describes mechanistic research focus across decades.
Median Cohort	Summary Statistic in Cohort Analyses	Appears in abstract-level signals of methodological layer.
MeSH	Medical Subject Headings	Controlled vocabulary in PubMed employed for precise field-restricted searching.
Metadata	Supplementary Bibliographic Descriptors	Retained for reproducibility and cross-referencing (authors, year, source title, etc.).
MicroRNAs/Cytokines	Small Regulatory RNAs/Signaling Proteins	Linked to radiation toxicity and inflammatory responses in prostate cancer.
MMRd	Mismatch Repair Deficiency	Genetic defect leading to hypermutation; associated with increased immunogenicity and favorable prognosis.
Model	Computational or Predictive Model	Refers to mathematical frameworks (e.g., dose-response, LQ, biological modeling).
Modularity	Measure of Community Segregation; Community-Segregation Index	Low modularity indicates overlapping thematic communities; very low value (≈0.02) shows themes overlap strongly with one weakly connected component.
MOUSE	Animal Model	Indicates in vivo preclinical testing in mice (*Topic_7a*).
Multi-Omic Biomarkers	Combined Genomic, Transcriptomic, and Proteomic Indicators	Used to stratify patients and personalize treatment strategies.
Multilingual Biomedical Corpora	Multi-Language Scientific Datasets	Describes the heterogeneity of textual sources posing semantic-alignment challenges.
n	Sample Size	Number of text pairs analyzed per class (Class 1: n = 6; Class 2: n = 6; Class 3: n = 18; Class 4: n = 4; Class 5: n = 3).
NER	Nucleotide Excision Repair	DNA repair process that removes bulky helix-distorting lesions; important for radiation and oxidative stress responses.
NER/SER/DER Metrics	Nucleotide Excision Repair/Sensitization Enhancement Ratio/Dose Enhancement Ratio	Quantitative parameters for evaluating biological amplification of radiation effects.
NHEJ	Non-Homologous End Joining	Fast, error-prone DNA repair mechanism; hyperactivation linked to radioresistance.
no_below = 2–5 /no_above = 0.5	Frequency-Trimming Parameters	Filter words appearing in fewer than 2–5 articles or in >50 % of texts, ensuring vocabulary stability.
Node/Edge	Vertex/Connection	Represent keywords and their co-occurrence relations.
Normal	Normal Tissue	Opposed to tumor tissue; represents non-targeted biological matter whose protection is critical in treatment planning.
Normalization/Standardization	Data-Cleaning Processes	Harmonized author, title, and metadata formats across databases before merging.
NSCLC	Non-Small Cell Lung Cancer	Disease context in studies on radiotherapy benefits.
NSMP	No Specific Molecular Profile	Endometrial cancer subtype lacking defined genomic markers; used for risk-based treatment calibration.
O	Overall (e.g., Overall Survival—OS)	Shortened reference to clinical endpoints (*Topic_3a*); likely from expressions such as “overall survival.”
Offline Analyses	Local Computation Without Internet Connectivity	Performed using Llama-3 for data security, sovereignty, and reproducibility.
Omics Data	Integrated Genomic, Transcriptomic, Proteomic Datasets	Used across classes for multi-scale modeling of tumor behavior.
Oncology	Medical Field of Cancer Diagnosis and Treatment	Provides the clinical component in the interdisciplinary framework.
OpenAI	Artificial Intelligence Research Organization	Developer of ChatGPT; referenced for model attribution.
OS	Overall Survival	Proportion of patients alive at specified follow-up times (1/3/5 years) after radiotherapy.
P	p-value (Statistical Significance)	Appears in *Topic_3a* and *4a*; denotes statistical significance in clinical or survival analyses.
PaperProcessor	LLM-Guided Semantic Extraction Script	Pipeline component for document-level summarization and tagging.
Parameter/Radiobiological	Model Coefficients/Domain Qualifier	Signal the quantitative calibration layer in abstracts.
Particle Therapy	Proton or Heavy-Ion Radiation Treatment	High-precision, high-LET modality for deep or radioresistant tumors.
passes = 10	Number of LDA Training Iterations	Controls convergence during topic-model optimization.
Pathway	Biological Pathway	Indicates molecular signaling cascades affected by radiation (e.g., DNA damage-repair pathways).
Pearson Correlation Coefficient	Statistical Measure (r); Statistical Correlation Index	Indicates robustness of network stability across runs (r > 0.9); confirms stability of node/community ordering between directed and undirected graphs.
PENt	Programa de Engenharia da Nanotecnologia (Nanotechnology Engineering Program, COPPE/UFRJ)	Academic home of the AI and semantic-modeling pipeline described.
Peripheral Nodes	Lower-Degree Vertices at Network Edges	Terms like proton, stereotactic, imaging anchor technology subfields.
Personalized/Protocol	Individualized Plans/Standardized Procedures	Bridge terms in co-occurrence graphs linking themes.
PFS	Progression-Free Survival	Interval during which a patient remains free from tumor progression or recurrence.
PLAN/DOS	Treatment Planning and Dosimetry	In *Topic_7a*, “plan” and “dos” (truncated for dose or dosimetry) refer to planning and dose-distribution modeling.
PNG	Portable Network Graphics	Raster format for graphical visualization outputs.
POLE	DNA Polymerase Epsilon (Mutated Subtype)	Mutation defining a favorable genomic subtype in endometrial cancer; used in precision oncology stratification.
PORTEC-3/PORTEC-4	Post-operative Radiation Therapy for Endometrial Carcinoma Trials	Landmark clinical studies incorporating molecular subtyping into radiotherapy decision frameworks.
Precision Oncology	Data-Driven Personalized Cancer Care; Data-Driven Personalized Cancer Treatment	Cross-cutting theme linking biomarkers to clinical protocols; integrates molecular profiling and computational modeling to guide clinical decisions.
Precision Radiotherapy	Data-Guided, Patient-Specific Treatment Framework	Conceptual endpoint of the integrated ecosystem.
Precision Radiotherapy Corpus	Consolidated Dataset	Final ≈ 45 deduplicated records informing thematic and network analyses.
Precision Radiotherapy Implementation Plan (PRIP)	Operational Framework; AI-Derived Operational Framework	Practical output of the study—semantic and molecular insights into five actionable clinical classes; translates computational and molecular insights into clinical strategies.
Preclinical	Preclinical Study; Experimental (Non-Clinical) Phase	Experimental phase prior to human clinical trials; linked to animal or in vitro testing (*Topic_5a*); denotes laboratory or animal studies preceding human trials.
Prediction/Response/Normal	High-Centrality Nodes	“Semantic hinges” connecting mechanistic and clinical themes in the supergraph.
Prediction Models	Computational or Statistical Outcome-Forecasting Tools	Represent the data-driven dimension emerging after 2010.
Predictive Systems	AI-Based Forecasting Models	Refer to evolving models that anticipate treatment response and guide dynamic planning.
Progression-Free Interval	Time to Recurrence	Quantitative measure for disease stability post-therapy.
Proteomics	Large-Scale Protein Analysis	Identifies functional markers of radioresistance and therapeutic targets.
Proton/Ion Beams	Particle-Beam Radiotherapy Modalities	Core of hadron-therapy and dose-delivery-physics studies.
Proton Therapy	Particle-Based Radiotherapy; Proton Therapy; Charged-Particle Radiation Modality	Advanced technique using proton beams for accurate dose delivery with minimal collateral damage; part of the technological wave correlated with publication surge.
Public Health Integration	Application of Precision Frameworks to Health-System Policies	Links AI-guided radiotherapy models to equity and access.
PubMed	U.S. National Library of Medicine Database (MEDLINE)	Primary biomedical source queried with MeSH and free-text terms; provided ≈ 66% of final records.
PubMed, Scopus, Web of Science (WoS)	Scientific Databases	Used for bibliometric data collection and harmonization.
Quantitative Metrics	Numerical Parameters or Indicators	Include dose, LET, RBE, similarity scores, or statistical validation values supporting model precision.
RAD51/BRCA1	DNA Repair Genes	Over-expression correlates with radioresistance; potential biomarkers for therapeutic targeting.
RAD51, PARP1, CHK1, MAPK15	Key DNA Repair/Stress Response Proteins	Biomarkers and therapeutic targets integrated into proteomic and genomic modeling layers.
Radiat/Cell/Dose/Tumor/Patient/Cancer/Radiobiolog	High-Frequency Lexical Stems	Most recurrent words in the processed corpus; define thematic backbone.
Radiobiological	Related to Radiation Biology	Describes models or parameters linking radiation dose to biological effects.
Radiobiological Integration	Coupling Biological Models and Clinical Protocols	Incorporates biological parameters (e.g., radiosensitivity indices) into planning.
Radiobiological Modeling	Quantitative Biological Modeling of Dose–Response	Used to predict tissue effects and optimize treatment.
Radiobiology	Study of Biological Effects of Ionizing Radiation	Core discipline analyzed alongside radiotherapy and oncology.
Radiomics	Quantitative Image-Feature Extraction	Supports predictive modeling and risk assessment integrated into adaptive radiotherapy.
Radioresistance Mechanisms	Molecular and Cellular Resistance Pathways	Refers to signaling and repair mechanisms that make tumor cells less responsive to radiation.
Radiotherapy (RT)	Use of Ionizing Radiation to Treat Cancer	Central disciplinary axis in all queries.
RadRes	Radiation Research (Journal)	Appears in a title summarizing 75 years of the field.
random_state = 42	Random-Seed Setting	Ensures reproducible topic-model results.
Rate (Dose-Rate)	Dose Per Unit Time	Critical radiobiological variable, particularly relevant for FLASH-RT and LDR contexts.
RBE	Relative Biological Effectiveness	Quantifies biological potency of one radiation type relative to another.
Repair	DNA or Cellular Repair	Biological process restoring damaged DNA or cellular integrity after irradiation.
Reproducibility	Methodological Consistency; Methodological Standards	Refers to achieving consistent quantitative and terminological results across independent AI systems; ensures that pre-processing and modeling steps can be independently verified.
RESPONSE	Biological or Clinical Response	Describes radiation-induced effects, gene-expression responses, or treatment outcomes.
Risk/Volume	Dosimetric and Anatomical Covariates	Terms indicating planning and outcome modeling foci.
RSI	Radiosensitivity Index	Genomic predictor of individual radiosensitivity used for dose calibration.
RT	Radiotherapy	Appears in several topics and class definitions.
RTOG	Radiation Therapy Oncology Group	U.S. co-operative group providing standardized toxicity and outcome reporting criteria.
SARS-CoV-2/COVID-19	Virus/Disease	Examined for potential changes in individual radiation sensitivity.
SBRT	Stereotactic Body Radiotherapy	Highly conformal, high-precision modality for small or lung tumors; advanced form of external beam radiotherapy delivering high-precision doses to small tumor volumes (directly referenced in *Topic_4t*).
Scopus	Elsevier’s Multidisciplinary Abstract and Citation Database	Used for automated retrieval of bibliographic records on radiotherapy, radiobiology, and oncology.
Scopus/PubMed/Web of Science (WoS)	Major Bibliographic Databases	Primary data sources for corpus construction (1964–2025); used for refining, validating, and harmonizing corpus selection.
SD/±	Standard Deviation	Expresses the dispersion of cosine-similarity values within each class.
Semantic Supergraph Analysis	Advanced Network Modeling Approach	Suggested future technique for identifying underexplored or emerging clusters within the scientific corpus.
Stereotactic Body Radiotherapy (SBRT)	High-Precision, Image-Guided Radiation Technique	Marks transition to modern radiotherapy (post-2010 growth phase).
Stopword Filtering	Removal of High-Frequency Function Words	Biomedical-specific list applied before modeling.
Supergraph	Global Integrated Co-Occurrence Network	Encompasses molecular, clinical, and technological domains.
Supergraph Inset	Central Visual in Figure 1	Depicts term relationships with colored weighted edges (blue = weak, red = strong).
SURVIVAL	Cellular or Patient Survival	Appears in Topics 1a and 3a, referring to both cell-survival assays and patient outcome metrics.
SUS/Unified Health System (SUS)	Sistema Único de Saúde (Brazil’s Unified Health System)	Framework for pilot implementation, ensuring scalability, equity, and cost-effectiveness; Brazil’s public-health system alignment target.
SVG	Scalable Vector Graphics	Visualization format for network maps.
t	Titles Corpus	The suffix “t” in *Topic_0t*–*Topic_9t* designates topics derived from article titles rather than abstracts.
Table 3/Figure 4	Plan Visualization Elements	Table summarizes indicators; figure visualizes network linking plan classes, health metrics, and strategic goals.
TARGET	Radiation Target Volume	Anatomical or biological region receiving prescribed radiation dose (*Topic_7a*).
TARGETED (THERAPY)	Molecularly Targeted Therapy	Used in *Topic_5a*; describes agents designed to act on specific molecular pathways.
TCGA	The Cancer Genome Atlas	Genomic classification system defining molecular subtypes across cancers; applied for adaptive radiotherapy.
TCP/NTCP	Tumor Control Probability/Normal Tissue Complication Probability	Radiobiological models estimating treatment success vs. toxicity.
Temporal Evolution Curve	Publication-Frequency Over Time	Depicts output growth and term-usage expansion (sharp post-2010 increase).
Term-Frequency Curve	Temporal Plot of Keyword Occurrence	Used to link bibliometric evolution to network topology.
TF-IDF	Term Frequency–Inverse Document Frequency	Weighting scheme representing articles numerically for cosine-similarity computation; emphasizes discriminative words.
Therap/Radiotherapi (Radiotherapy Lemmatized Form)	Lemmatized Word Stems	Generated during text preprocessing before word-cloud and LDA analysis.
Therapeutic Window	Efficacy–Toxicity Balance	Keyword guiding selection of translationally relevant articles.
Tissue/Normal	Biological Material (Tumor vs. Normal)	Distinction between targeted tumor tissue and healthy tissue, relevant to toxicity models (*Topic_9a*).
TITLE-ABS-KEY	Title–Abstract–Keyword Query Field in Scopus	Syntax specifying that Boolean terms be searched in all three metadata fields.
*Topic_0a*–*Topic_9a*	Abstract-Based LDA Topics	Capture methodological and biological dimensions (e.g., FLASH-RT, RBE, immunotherapy).
*Topic_0t*–*Topic_9t*	Title-Based LDA Topics	Identify clusters such as tumor sites, radiation types, or molecular mechanisms; each topic represents a statistically derived keyword cluster from titles.
Toxicity	Measure of Treatment-Induced Adverse Effects	One of the outcome indicators for therapy optimization.
TP53/p53abn	Tumor Protein 53/Abnormal TP53 Subtype	Gene regulating cell cycle and apoptosis; mutation indicates poor prognosis in endometrial and cervical cancers.
Translational Continuity	Bridging Experimental and Clinical Domains	Describes how findings progress from bench to bedside within a single analytical cycle.
Translational Publication Types/Translational Trials	Research Bridging Lab and Clinic/Studies Bridging Lab Findings and Clinical Implementation	Filters applied in PubMed to maximize relevance; reflected by terms “clinic,” “trial,” “meta,” “evalu.”
Transparency	Disclosure of AI Tool Use	Explicitly described to comply with academic-integrity and reproducibility standards.
Two-Dimensional Thematic Landscape	Dual-Axis Conceptual Map	Represents integration of clinical and molecular radiotherapy research.
UFRJ	Universidade Federal do Rio de Janeiro (Federal University of Rio de Janeiro)	Institutional affiliation of multiple authors.
Undirected, Thresholded Version	Simplified Graph with Bidirectional Edges and Minimum-Weight Cutoff	Used to test robustness of results.
VHL	von Hippel–Lindau Gene	Mutation induces pseudohypoxia and pro-survival signaling in renal carcinoma.
Vitro (from in vitro)	In Vitro Studies	Laboratory studies conducted outside living organisms, often in cell cultures, used to study radiation-response mechanisms.
VOSviewer	Visualization of Similarities Viewer	Software for bibliometric and co-occurrence network visualization.
Voxel/Voxel-Based Analysis/Voxel-Based Mapping/Voxel-Level Analytics	Volumetric Pixel and Spatial Dose–Response Modeling Methods	Voxel = smallest unit in 3D medical imaging and dosimetry; used for dose/response mapping and adaptive planning; links radiobiological effects to 3-D anatomical regions for spatially resolved optimization.
Weakly Connected Component	Subgraph with at Least One Directional Path Between All Nodes	Indicates overall semantic unity of the dataset.
Web of Science (WoS)	Clarivate’s Multidisciplinary Citation Index	Used with direct keyword search to complement PubMed and Scopus coverage.
Weighted Degree	Sum of Edge Weights for a Node	Counts total co-occurrence frequency rather than binary presence.
Word Cloud	Frequency-Scaled Visual Representation of Keywords	Summarizes lexical prominence from titles and abstracts.
α-Parameter	Linear Component of the LQ Model	Represents cell-killing probability per unit dose in radiobiological modeling.
α/β Values	Linear–Quadratic Model Parameters	Describe tissue-specific radiation response; used in pediatric and comparative modeling.
γH2AX	Phosphorylated Histone H2AX	Biomarker of DNA double-strand breaks; persistence indicates inefficient repair.

## Appendix A. Topics from Titles (t)

***Topic_0t*** = 0.090 × “*cancer*” + 0.050 × “*radiotherapy*” + 0.049 × “*patient*” + 0.038 × “*head*” + 0.037 × “*neck*” + 0.022 × “*breast*” + 0.018 × “*prostate*” + 0.013 × “*toxicity*” + 0.011 × “*advanced*” + 0.010 × “*treatment*” (A1)

***Topic_1t*** = 0.075 × “*radiobiology*” + 0.040 × “*radiotherapy*” + 0.036 × “*clinical*” + 0.031 × “*radiation*” + 0.026 × “*therapy*” + 0.019 × “*radiobiological*” + 0.016 × “*treatment*” + 0.015 × “*oncology*” + 0.013 × “*perspective*” + 0.010 × “*new*” (A2)

***Topic_2t*** = 0.050 × “*cancer*” + 0.042 × “*radiotherapy*” + 0.021 × “*patient*” + 0.021 × “*breast*” + 0.018 × “*carcinoma*” + 0.014 × “*treatment*” + 0.013 × “*prostate*” + 0.012 × “*cell*” + 0.010 × “*esophageal*” + 0.009 × “*comparison*” (A3)

***Topic_3t*** = 0.097 × “*radiation*” + 0.046 × “*oncology*” + 0.023 × “*therapy*” + 0.022 × “*biology*” + 0.020 × “*mechanism*” + 0.014 × “*molecular*” + 0.011 × “*medical*” + 0.011 × “*injury*” + 0.011 × “*effect*” + 0.011 × “*clinical*” (A4)

***Topic_4t*** = 0.058 × “*stereotactic*” + 0.033 × “*body*” + 0.031 × “*therapy*” + 0.029 × “*lung*” + 0.028 × “*cancer*” + 0.028 × “*radiotherapy*” + 0.027 × “*cell*” + 0.025 × “*radiosurgery*” + 0.023 × “*tumor*” + 0.021 × “*radiation*” (A5)

***Topic_5t*** = 0.044 × “*radiation*” + 0.042 × “*cancer*” + 0.040 × “*therapy*” + 0.024 × “*cell*” + 0.021 × “*tissue*” + 0.020 × “*normal*” + 0.018 × “*beam*” + 0.018 × “*ion*” + 0.013 × “*proton*” + 0.013 × “*effect*” (A6)

***Topic_6t*** = 0.039 × “*radiotherapy*” + 0.029 × “*tumor*” + 0.020 × “*cancer*” + 0.016 × “*radiation*” + 0.015 × “*therapy*” + 0.015 × “*patient*” + 0.015 × “*brain*” + 0.012 × “*model*” + 0.011 × “*imaging*” + 0.011 × “*preclinical*” (A7)

***Topic_7t*** = 0.049 × “*tumor*” + 0.034 × “*cell*” + 0.026 × “*dna*” + 0.023 × “*repair*” + 0.022 × “*factor*” + 0.019 × “*cancer*” + 0.014 × “*lung*” + 0.013 × “*targeting*” + 0.013 × “*radiotherapy*” + 0.013 × “*pathway*” (A8)

***Topic_8t*** = 0.093 × “*cell*” + 0.039 × “*human*” + 0.028 × “*effect*” + 0.023 × “*expression*” + 0.022 × “*radiation*” + 0.018 × “*gene*” + 0.017 × “*carcinoma*” + 0.016 × “*line*” + 0.015 × “*vitro*” + 0.014 × “*irradiation*” (A9)

***Topic_9t*** = 0.089 × “*dose*” + 0.033 × “*radiotherapy*” + 0.030 × “*rate*” + 0.029 × “*brachytherapy*” + 0.018 × “*high*” + 0.016 × “*radiobiology*” + 0.016 × “*prostate*” + 0.015 × “*model*” + 0.013 × “*linear*” + 0.012 × “*radiobiological*” (A10)

## Appendix B. Topics from Abstracts (a)

***Topic_0a*** = 0.033 × “*model*” + 0.013 × “*dose*” + 0.012 × “*tumor*” + 0.012 × “*imaging*” + 0.009 × “*flash*” + 0.009 × “*feature*” + 0.008 × “*clinical*” + 0.008 × “*image*” + 0.007 × “*parameter*” + 0.006 × “*volume*” (A11)

***Topic_1a*** = 0.070 × “*cell*” + 0.021 × “*tumor*” + 0.014 × “*effect*” + 0.013 × “*dna*” + 0.009 × “*repair*” + 0.009 × “*response*” + 0.009 × “*damage*” + 0.008 × “*line*” + 0.007 × “*mechanism*” + 0.006 × “*survival*” (A12)

***Topic_2a*** = 0.033 × “*proton*” + 0.025 × “*ion*” + 0.022 × “*rbe*” + 0.021 × “*beam*” + 0.016 × “*particle*” + 0.014 × “*carbon*” + 0.014 × “*energy*” + 0.014 × “*biological*” + 0.013 × “*therapy*” + 0.012 × “*photon*” (A13)

***Topic_3a*** = 0.044 × “*patient*” + 0.016 × “*rt*” + 0.015 × “*survival*” + 0.015 × “*tumor*” + 0.011 × “*p*” + 0.008 × “*surgery*” + 0.008 × “*local*” + 0.008 × “*rate*” + 0.008 × “*overall*” + 0.007 × “*o*” (A14)

***Topic_4a*** = 0.056 × “*patient*” + 0.019 × “*toxicity*” + 0.015 × “*p*” + 0.011 × “*breast*” + 0.010 × “*risk*” + 0.010 × “*grade*” + 0.010 × “*gy*” + 0.010 × “*month*” + 0.008 × “*sbrt*” + 0.008 × “*treated*” (A15)

***Topic_5a*** = 0.033 × “*study*” + 0.028 × “*therapy*” + 0.022 × “*clinical*” + 0.017 × “*trial*” + 0.015 × “*preclinical*” + 0.012 × “*tumor*” + 0.012 × “*immunotherapy*” + 0.011 × “*targeted*” + 0.010 × “*agent*” + 0.010 × “*promising*” (A16)

***Topic_6a*** = 0.023 × “*expression*” + 0.019 × “*cell*” + 0.017 × “*gene*” + 0.012 × “*response*” + 0.012 × “*patient*” + 0.011 × “*tumor*” + 0.008 × “*level*” + 0.007 × “*irradiation*” + 0.007 × “*protein*” + 0.006 × “*blood*” (A17)

***Topic_7a*** = 0.026 × “*dose*” + 0.023 × “*gy*” + 0.020 × “*irradiation*” + 0.014 × “*cell*” + 0.013 × “*tumor*” + 0.012 × “*plan*” + 0.011 × “*dos*” + 0.009 × “*day*” + 0.009 × “*target*” + 0.008 × “*mouse*” (A18)

***Topic_8a*** = 0.016 × “*clinical*” + 0.012 × “*therapy*” + 0.012 × “*oncology*” + 0.008 × “*radiobiology*” + 0.007 × “*patient*” + 0.006 × “*right*” + 0.006 × “*reserved*” + 0.006 × “*medical*” + 0.006 × “*development*” + 0.006 × “*elsevier*” (A19)

***Topic_9a*** = 0.039 × “*dose*” + 0.020 × “*tumor*” + 0.014 × “*fraction*” + 0.013 × “*tissue*” + 0.012 × “*model*” + 0.012 × “*effect*” + 0.011 × “*gy*” + 0.009 × “*normal*” + 0.008 × “*rate*” + 0.008 × “*cell*” (A20)
